# Race and Ethnicity, Lifestyle, Diet, and Survival in Patients With Prostate Cancer

**DOI:** 10.1001/jamanetworkopen.2024.60785

**Published:** 2025-02-26

**Authors:** Anqi Wang, Erin L. Van Blarigan, Iona Cheng, June M. Chan, Peggy Wan, Song-Yi Park, Wei Xiong, Ann S. Hamilton, Fei Chen, Loic Le Marchand, Lynne R. Wilkens, David V. Conti, Stacey A. Kenfield, Christopher A. Haiman

**Affiliations:** 1 Center for Genetic Epidemiology, Department of Population and Public Health Sciences, Keck School of Medicine, University of Southern California, Los Angeles; 2 Department of Epidemiology, Harvard T.H. Chan School of Public Health, Boston, Massachusetts; 3 Department of Urology, University of California, San Francisco; 4 Department of Epidemiology and Biostatistics, University of California, San Francisco; 5 Helen Diller Family Comprehensive Cancer Center, University of California, San Francisco; 6 Population Sciences in the Pacific Program, University of Hawaii Cancer Center, Honolulu

## Abstract

**Importance:**

Prostate cancer (PCa) remains a leading cause of cancer-related death among men in the US.

**Objective:**

To evaluate the association of healthy lifestyle and dietary behaviors with survival after a nonmetastatic PCa diagnosis in a multiethnic population.

**Design, Setting, and Participants:**

This prospective cohort study was conducted among men aged 45 to 75 years enrolled between 1993 and 1996 in the Multiethnic Cohort study. Participants with nonmetastatic PCa completed a questionnaire after diagnosis (2003-2008) and were followed up until death or loss to follow-up. Data were analyzed from January 10, 2023, to May 20, 2024.

**Exposures:**

Lifestyle and dietary patterns were assessed after diagnosis using 3 PCa behavior scores and 13 dietary indices (4 prioritized scores: the Healthy Eating Index–2015, Healthful Plant-Based Diet Index, Dietary Inflammatory Index, and Empirical Dietary Index for Hyperinsulinemia).

**Main Outcomes and Measures:**

Cox proportional hazards models were used to evaluate multivariable-adjusted associations of each PCa behavior score with all-cause, cardiovascular disease (CVD), and PCa-specific mortality.

**Results:**

A total of 2603 men with nonmetastatic PCa (mean [SD] age, 69.6 [7.1] years) were followed up, and 1346 deaths were documented, including 356 (24.6%) from CVD and 197 (14.6%) from PCa. The median (IQR) follow-up was 10.9 (IQR, 6.8-12.7) years from questionnaire return and 14.5 (IQR, 11.8-18.0) years from diagnosis. The 2021 PCa Behavior Score was associated with reduced risks of all-cause (hazard ratio [HR] per point, 0.69; 95% CI, 0.63-0.77) and CVD-related (HR, 0.67; 95% CI, 0.56-0.79) mortality. This score was also associated with a lower risk of PCa-specific mortality among African American men (HR, 0.46; 95% CI, 0.24-0.88) but not in the other racial and ethnic groups. Comparing quintile 5 (highest score) with 1 (lowest score), the Empirical Dietary Index for Hyperinsulinemia was positively associated with all-cause (HR, 1.37; 95% CI, 1.02-1.84) and CVD-related (HR, 1.96; 95% CI, 1.15-3.33) mortality, whereas the Healthful Plant-Based Diet Index was associated with a reduced risk of all-cause (HR, 0.75; 95% CI, 0.58-0.97); findings for CVD-related mortality were not statistically significant (HR, 0.67; 95% CI, 0.44-1.03). No associations were found between lifestyle or dietary patterns and PCa mortality.

**Conclusions and Relevance:**

In this multiethnic cohort of patients with nonmetastatic PCa, healthier lifestyles were associated with improved overall survival but not with PCa-specific survival. Given the predominance of non–PCa-specific deaths, these findings support the need for health behavior counseling to treat comorbidities in men with PCa.

## Key Points

**Question:**

Are healthier lifestyle and dietary behaviors after a diagnosis of nonmetastatic prostate cancer associated with improved long-term survival outcomes among patients from diverse racial and ethnic groups?

**Findings:**

In this cohort study of 2603 patients with nonmetastatic prostate cancer followed up for a median of 10.9 years, healthier lifestyle and dietary patterns were associated with reduced all-cause and cardiovascular disease mortality.

**Meaning:**

These findings suggest that health behavior counseling may enhance overall long-term survival in men with nonmetastatic prostate cancer, highlighting its potential importance in clinical management.

## Teaser

This cohort study evaluates the association of healthy lifestyle and dietary behaviors with survival after a diagnosis of nonmetastatic prostate cancer in a multiethnic sample of US men.

## Introduction

Epidemiologic studies of postdiagnostic modifiable factors and prostate cancer (PCa) prognosis remain inconclusive.^1,2,3^ Evidence suggests that postdiagnosis smoking and high-fat dairy intake are associated with increased all-cause and PCa-specific mortality risk among patients with PCa,^1,2^ whereas physical activity has been associated with benefit, such as improved physical functioning and reduced PCa-specific mortality.^2,3^ Obesity has been associated with poor prognosis, although this association is attenuated when accounting for pathologic characteristics.^1,4^ Specific dietary components, such as tomatoes^5,6,7^ and cruciferous vegetables,^8^ have shown potential for improving PCa survival, though findings lack replication.^2^ Furthermore, behavioral scores comprising multiple risk factors have been developed to examine an aggregate association with PCa outcomes. Previous studies of postdiagnostic lifestyle scores in White populations have indicated that a lifestyle of nonsmoking, a healthy body mass index (BMI), vigorous physical activity, and a healthy diet may improve PCa progression and survival.^9,10^

In addition to overall lifestyle patterns, dietary indices have been developed to evaluate the influence of specific dietary patterns on health outcomes.^11^ Indices that quantify the inflammatory and insulinemic potential of diets, empirically developed on the basis of blood biomarkers, have been found to be associated with risk of advanced PCa and disease progression.^12,13^ Additionally, scores that categorize eating styles across populations, such as the Healthy Eating Index (HEI) and plant-based diet indices (PDIs), have been associated with lower risks of total and lethal PCa,^14,15^ as well as disease progression.^16,17^ However, evidence of the postdiagnosis influence of these dietary patterns is limited in racially and ethnically diverse populations.

Considering the variation in the prevalence of lifestyle and dietary patterns across different racial and ethnic groups, it is important to determine whether lifestyle scores developed and associated with health outcomes in White populations generalize to other racial and ethnic groups. In this study, we evaluated the associations between previously reported lifestyle and dietary scores and risk of PCa, cardiovascular disease (CVD), and overall mortality in a prospective multiethnic cohort of men initially diagnosed with nonmetastatic PCa.

## Methods

### Study Population

The Multiethnic Cohort (MEC) study is a prospective cohort that enrolled more than 215 000 participants aged 45 to 75 years between 1993 and 1996 in California (mainly Los Angeles) and Hawaii.^18^ The study was approved by the institutional review boards at the University of Hawaii and the University of Southern California. Participants provided written informed consent. This study followed the Strengthening the Reporting of Observational Studies in Epidemiology (STROBE) reporting guideline for cohort studies.

Participants who self-reported their race and ethnicity as African American, Japanese American, Latino, Native Hawaiian, or White were eligible for inclusion in the MEC. The racial and ethnic categories were determined by the investigators at the time the MEC was initiated. Participants were identified through their state driver’s license, supplemented by their voter registration in Hawaii and the Health Care Financing Administration in California.^18^ At baseline (QX1), participants completed a self-administrated questionnaire that covered demographic information, medical history, medication use, and lifestyle habits (eg, smoking status, physical activity) (henceforth, referred to as QX1). A validated quantitative food frequency questionnaire was used to assess dietary habits during the previous year, which included the frequency of intake and usual quantity in a serving of more than 180 food items.^19^ A repeat of the baseline survey was conducted in 2003-2008 (QX3) to update medical conditions and lifestyle and dietary behaviors.

### PCa and Cause of Death Ascertainment

To study postdiagnostic lifestyle factors, we focused on men with PCa diagnosed between QX1 and QX3 (1993-2008). Patients with PCa were identified by linkage to the Surveillance, Epidemiology, and End Results California and Hawaii cancer registries. Data on date of diagnosis, stage at diagnosis, Gleason grade, and first course of treatment were obtained from the registries. Dates and primary causes of death were ascertained from death certificate files in Hawaii and California, supplemented by the National Death Index. Mortality information was completed through December 31, 2017. We categorized the primary causes of death into different groups based on *International Statistical Classification of Diseases, Tenth Revision* codes (eTable 1 in Supplement 2).

### Inclusion and Exclusion Criteria

As shown in eFigure 1 in Supplement 1, 6764 men had a diagnosis of PCa between QX1 and QX3. We excluded 310 for implausible dietary intake at QX1, 787 for missing stage and grade information or metastatic disease at diagnosis, and 3064 for incomplete QX3, resulting in a final analytic sample of 2603. Compared with excluded participants, those included were generally younger at diagnosis (approximately 2 years), had higher education levels, were more likely to self-report White or Japanese American race, and had a family history of PCa. No significant differences in disease stage or grade at diagnosis were observed.

### Healthy Lifestyle Scores

We used the 2021 PCa Behavior Score (2021 Score) to assess postdiagnostic lifestyle patterns specific to PCa. This index was developed for PCa-related mortality based on lifestyle factors, including BMI, total physical activity, and smoking status.^9^ We also evaluated the 2021 PCa Behavior Score Including Diet (2021 Score + Diet), which additionally incorporates dietary intake of saturated fat, whole milk, alcohol, and processed meat.^9^ Furthermore, we evaluated the 2015 PCa Behavior Score (2015 Score), an index developed on the basis of risk factors for incident lethal PCa (metastatic or fatal disease), including BMI; physical activity; smoking status; and dietary intake of tomatoes, high-fat fish, and processed meats.^20^ All aforementioned factors were assessed at QX1 (before diagnosis) and QX3 (after diagnosis). We computed the lifestyle scores by summing the scores of each factor as described in eTable 2 in Supplement 2.^9,20^ For individuals with missing data on a specific factor at QX3, the value of that factor from QX1 was used (n = 375).

### Dietary Quality Indices

We evaluated 13 predefined dietary quality indices^21,22,23,24,25,26,27^ (detailed in eTable 3 in Supplement 2). Each dietary index was categorized into 5 groups based on the quintile distribution of the scores, with the quintiles determined among men in the entire cohort at QX1, reflecting general population distributions. Because of the extensive number of indices examined, we prioritized 4 dietary indices, the HEI-2015, Healthful Plant-Based Diet Index (hPDI), Energy-Adjusted Dietary Inflammatory Index (E-DII), and Empirical Dietary Index for Hyperinsulinemia (EDIH), which we selected based on their correlation with the majority of other indices (eFigure 2 in Supplement 1). The results for the remaining 9 dietary patterns are detailed in Supplement 1 and Supplement 2.

### Statistical Analysis

The data analysis was performed from January 10, 2023, to May 20, 2024. We used Cox proportional hazards regression models with age as the time metric to estimate hazard ratios (HRs) and 95% CIs for the association of each lifestyle and dietary index measured at QX3 with all-cause, PCa-specific, and CVD-related mortality. Follow-up was calculated from the date at QX3 to death, loss to follow-up, or censoring on December 31, 2017. We examined all indices both as continuous (per 1-point or SD increase) and as categorical variables.

For each association test, we adjusted in model 1 for age at diagnosis, race and ethnicity, education, family history of PCa, and daily energy intake from QX3. Model 2 further adjusted for PCa stage (localized or regional) and grade (Gleason ≥8 or ≤7) at diagnosis, treatment at diagnosis (surgery, hormone therapy, chemotherapy, or radiation therapy), and other modifiable factors from QX3 if not included in the score calculation (ie, percentage of calories from saturated fat intake, whole milk intake, alcohol consumption, high-fat fish intake, tomato intake, and processed meat intake when examining lifestyle scores; BMI; smoking; and physical activity when examining dietary indices). All models were stratified by calendar year at diagnosis.

We performed analyses in the overall population and stratified by race and ethnicity. We estimated heterogeneity among racial and ethnic groups using the restricted maximum likelihood method in a random-effects model via the *Q* statistic. As a sensitivity analysis, we stratified by tertiles of indices at QX1 (before diagnosis) to isolate the postdiagnostic effect of the indices on survival. Additionally, to account for the possibility of survival bias, we examined all men diagnosed with PCa between QX1 and QX3, regardless of their status at QX3 (n = 5667). We used a time-varying analysis. Participants entered the analysis at the date of PCa diagnosis. Scores were based on QX1 and updated to QX3 post diagnosis (if available). Scores at QX1 and QX3 were moderately to strongly correlated (correlation coefficient range, 0.37-0.73) (eFigure 2 in Supplement 1), which supports the use of QX1 scores as a reliable proxy for men missing QX3 data. Participants were then followed up to death or administrative censoring, whichever occurred first. All analyses considered a 2-tailed *P* < .05 to be statistically significant without applying a correction for multiple comparisons. The statistical analysis was performed using Stata, version 14.1 (StataCorp LLC) and R, version 4.0.0 (R Foundation).

## Results

We followed up 2603 men diagnosed with nonmetastatic PCa for a median of 10.9 years (IQR, 6.8-12.7 years) after questionnaire return (QX3) and a median of 14.5 years (IQR, 11.8-18.0 years) after diagnosis (mean [SD] age at diagnosis, 69.6 [7.1] years; 497 self-reporting as African American [19.1%], 754 as Japanese American [29.0%], 577 as Latino [22.2%], 129 as Native Hawaiian [5.0%], and 646 as White [24.8%] race and ethnicity). Demographic and clinical characteristics at diagnosis are shown in Table 1. At diagnosis, 2324 participants (89.3%) had localized cancer, and 1980 (76.1%) had a Gleason grade of at least 7; 855 participants (32.8%) underwent surgery, 1207 (46.4%) underwent radiation therapy, and 785 (30.2%) underwent hormone therapy. African American participants had the highest percentage of grade 7 or lower disease (85.3% [424 of 497 participants]) and the lowest percentage of first-course treatment received (74.8% [372 participants]). Of a total of 1346 deaths, 197 (14.6%) were from PCa. The most common other causes of death were CVD (356 participants [26.4%]), other cancer (281 participants [20.9%]), cerebrovascular disease (70 participants [5.2%]), and chronic pulmonary disease (46 participants [3.4%]) (eFigure 3 in Supplement 1).

**Table 1.** Characteristics of Men With Nonmetastatic PCa Diagnosed Between QX1 and QX3 in the MEC, Overall and by Race and Ethnicity (N = 2603)

Characteristic	Men, No. (%)					
	Total	African American	Japanese American	Latino	Native Hawaiian	White
No. of men	2603	497 (19.1)	754 (29.0)	577 (22.2)	129 (5.0)	646 (24.8)
Age at diagnosis, mean (SD), y	69.6 (7.1)	69.0 (7.1)	71.3 (7.0)	69.1 (6.6)	68.3 (6.6)	68.9 (7.6)
Education						
≤8th Grade	213 (8.2)	24 (4.8)	17 (2.3)	149 (25.8)	7 (5.4)	16 (2.5)
9th-12th Grade	759 (29.2)	139 (28.0)	286 (37.9)	173 (30.0)	58 (45.0)	103 (15.9)
Vocational school or some college	756 (29.0)	180 (36.2)	213 (28.2)	151 (26.2)	35 (27.1)	177 (27.4)
Graduated college or higher	850 (32.7)	147 (29.6)	236 (31.3)	94 (16.3)	26 (20.2)	347 (53.7)
Missing	25 (1.0)	7 (1.4)	2 (0.3)	10 (1.7)	3 (2.3)	3 (0.5)
Family history of PCa	305 (12.5)	64 (14.5)	78 (10.8)	64 (12.1)	12 (9.7)	87 (14.0)
Stage						
Localized	2324 (89.3)	448 (90.1)	685 (90.8)	489 (84.7)	118 (91.5)	584 (90.4)
Regional	279 (10.7)	49 (9.9)	69 (9.2)	88 (15.3)	11 (8.5)	62 (9.6)
Gleason grade						
≤7	1980 (76.1)	424 (85.3)	508 (67.4)	473 (82.0)	87 (67.4)	488 (75.5)
≥8	623 (23.9)	73 (14.7)	246 (32.6)	104 (18.0)	42 (32.6)	158 (24.5)
Primary treatment						
Surgery						
No	1468 (56.4)	319 (64.2)	460 (61.0)	290 (50.3)	68 (52.7)	331 (51.2)
Yes	855 (32.8)	172 (34.6)	166 (22.0)	285 (49.4)	36 (27.9)	196 (30.3)
Missing	280 (10.8)	6 (1.2)	128 (17.0)	2 (0.3)	25 (19.4)	119 (18.4)
Radiation therapy						
No	1394 (53.6)	332 (66.8)	301 (39.9)	397 (68.8)	50 (38.8)	314 (48.6)
Yes	1207 (46.4)	165 (33.2)	453 (60.1)	179 (31.0)	79 (61.2)	331 (51.2)
Missing	2 (0.1)	0	0	1 (0.2)	0	1 (0.2)
Hormone therapy						
No	1618 (62.2)	319 (64.2)	449 (59.5)	380 (65.9)	73 (56.6)	397 (61.5)
Yes	785 (30.2)	101 (20.3)	273 (36.2)	146 (25.3)	53 (41.1)	212 (32.8)
Missing	200 (7.7)	77 (15.5)	32 (4.2)	51 (8.8)	3 (2.3)	37 (5.7)
Chemotherapy						
No	2421 (93.0)	415 (83.5)	733 (97.2)	522 (90.5)	128 (99.2)	623 (96.4)
Yes	10 (0.4)	4 (0.8)	1 (0.1)	1 (0.2)	0	4 (0.6)
Missing	172 (6.6)	78 (15.7)	20 (2.7)	54 (9.4)	1 (0.8)	19 (2.9)
Received any primary treatment						
No	282 (10.8)	96 (19.3)	63 (8.4)	72 (12.5)	7 (5.4)	42 (6.5)
Yes	2164 (83.1)	372 (74.8)	637 (84.5)	495 (85.8)	109 (84.5)	552 (85.4)
Missing	157 (6.0)	29 (5.8)	54 (7.2)	10 (1.7)	13 (10.1)	52 (8.0)

Abbreviations: MEC, Multiethnic Cohort study; PCa, prostate cancer; QX1, baseline questionnaire; QX3, follow-up questionnaire (2003-2008).

Japanese American participants had the highest healthy lifestyle scores, with 23.6% (174 of 738) in the healthiest category of the 2021 Score, while African American men had the lowest, with 13.0% (60 of 463) in the least healthy category (eFigure 4 in Supplement 1). Participants’ postdiagnostic diets were generally healthier than their prediagnostic diets. African American and Native Hawaiian participants tended to have higher E-DII and EDIH scores, indicating more proinflammatory and proinsulinemic dietary patterns. In contrast, Japanese American participants reported diets with lower E-DII and higher hPDI scores, suggesting a greater consumption of plant-based foods.

**Figure 1.**
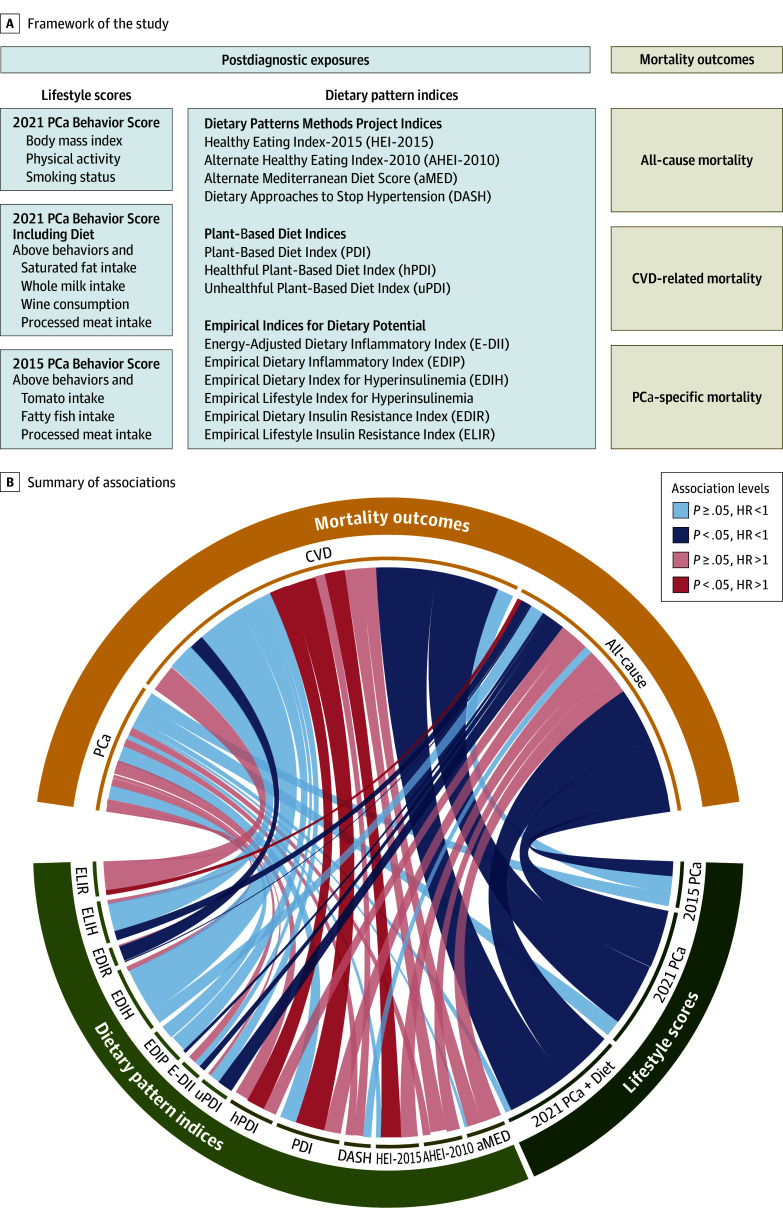
Framework and Associations of Lifestyle and Dietary Indices With Mortality Among Patients With Nonmetastatic Prostate Cancer (PCa) A. Dietary pattern indices are categorized into healthy (HEI-2015, AHEI-2010, aMED, DASH, PDI, hPDI) and adverse indices (uPDI, E-DII, EDIP, EDIH, ELIH, EDIR, ELIR) on the basis of their association with dietary health. B. Models were adjusted for age at diagnosis, education, race and ethnicity, family history of PCa, PCa stage, grade, treatments, total calorie intake at the follow-up questionnaire (2003-2008), and lifestyle or dietary factors not incorporated in the scores. Dietary scores are analyzed per standard deviation, while lifestyle scores are evaluated per point. The width of the links reflects the magnitude of the association between each healthy lifestyle score and dietary index with mortality, scaled by the absolute effect sizes (|log(HR)|) within a range from 0 to 1. For dietary scores of EDIP, EDIR, ELIH, ELIR, and uPDI, higher scores indicate dietary patterns associated with adverse health outcomes; conversely, higher scores on aMED, AHEI-2010, HEI-2015, DASH, and PDI correspond with adherence to dietary patterns conducive to positive health outcomes. CVD indicates cardiovascular disease; HR, hazard ratio.

The conceptual framework is summarized in Figure 1A, with an overview of the associations detailed in Figure 1B. Specifically, in the fully adjusted model, each 1-point increase in the 2021 Score was significantly associated with a reduction in all-cause mortality (HR per point, 0.69; 95% CI, 0.63-0.77) (Table 2; Figure 1B). Compared with participants in the lowest score category (0-1), those in the highest score category (3) had a 47% (95% CI, 32%-59%) reduction in all-cause mortality (*P* for trend < .001). Similarly, greater inverse associations were observed with the 2021 Score + Diet. The 2021 Score was also associated with a lower risk of CVD-related mortality (HR per point, 0.67; 95% CI, 0.56-0.79), with consistent results observed when incorporating diet. Risk reduction for PCa mortality was not statistically significant (HR per 1-point increase of the 2021 Score, 0.90; 95% CI, 0.69-1.18). No evidence of a linear trend was observed (*P* for trend = .66). When including diet in the score (2021 Score + Diet), risks improved for intermediate score categories (HR of 1.75-2.25 vs <1.50 points, 0.58; 95% CI, 0.35-0.97), although the linear outcome remained nonsignificant in all models. Similar patterns were observed for the 2015 Score (eTable 4 in Supplement 2). None of the individual lifestyle or dietary factors included in the score or dietary factors reported in previous studies were associated with PCa mortality in adjusted models (eTable 5 in Supplement 2).

**Table 2.** Associations Between Healthy Lifestyle Scores and Mortality Among Men With Nonmetastatic PCa in the MEC (N = 2603)

Variable	Healthy lifestyle score category^a^					*P* value for trend	Per 1-point increase	
							Data	*P* value
**All-cause mortality**								
2021 PCa Behavior Score	0-1	1.5	2	2.5	3	NA	NA	NA
No. at risk, men (person-y)	179 (1491)	394 (3434)	750 (7321)	796 (8062)	397 (3954)	NA	2571 (24 706)	NA
No. of events	126	245	370	357	181	NA	1321	NA
Model 1, HR (95% CI)^b^	1 [Reference]	0.88 (0.69-1.10)	0.58 (0.47-0.73)	0.52 (0.42-0.65)	0.51 (0.40-0.65)	<.001	0.68 (0.62-0.74)	<.001
Model 2, HR (95% CI)^c^	1 [Reference]	0.87 (0.69-1.09)	0.59 (0.47-0.74)	0.52 (0.42-0.66)	0.53 (0.41-0.68)	<.001	0.69 (0.63-0.77)	<.001
2021 PCa Behavior Score Including Diet	0-1.5	1.75-2.25	2.5-3	3.25-3.5	3.75-4	NA	NA	NA
No. at risk, men (person-y)	278 (2255	719 (6734)	1071 (10 615)	382 (3893)	121 (1210)	NA	2571 (24 706)	NA
No. of events	200	398	503	168	52	NA	1321	NA
Model 1, HR (95% CI)^b^	1 [Reference]	0.69 (0.57-0.83)	0.51 (0.43-0.61)	0.47 (0.37-0.58)	0.46 (0.33-0.64)	<.001	0.70 (0.64-0.76)	<.001
Model 2, HR (95% CI)^d^	1 [Reference]	0.68 (0.56-0.82)	0.52 (0.43-0.62)	0.46 (0.37-0.58)	0.49 (0.35-0.68)	<.001	0.70 (0.64-0.77)	<.001
**CVD-related mortality**								
2021 PCa Behavior Score	0-1.5	2	2.5	3	NA	NA	NA	NA
No. at risk, men (person-y)	573 (4924)	750 (7321)	796 (8062)	397 (3954)	NA	NA	2571 (24 706)	NA
No. of events	129	120	104	50	NA	NA	415	NA
Model 1, HR (95% CI)^b^	1 [Reference]	0.66 (0.50-0.86)	0.51 (0.38-0.67)	0.48 (0.33-0.67)	NA	<.001	0.63 (0.53-0.74)	<.001
Model 2, HR (95% CI)^c^	1 [Reference]	0.68 (0.52-0.90)	0.53 (0.40-0.71)	0.53 (0.37-0.76)	NA	<.001	0.67 (0.56-0.79)	<.001
2021 PCa Behavior Score Including Diet	0-1.5	1.75-2.25	2.5-3	3.25-4	NA	NA	NA	NA
No. at risk, men (person-y)	278 (2255)	719 (6734)	1071 (10 615)	503 (5103)	NA	NA	2571 (24 706)	NA
No. of events	65	137	154	59	NA	NA	415	NA
Model 1, HR (95% CI)^b^	1 [Reference]	0.71 (0.51-0.98)	0.49 (0.36-0.67)	0.40 (0.27-0.58)	NA	<.001	0.66 (0.57-0.77)	<.001
Model 2, HR (95% CI)^d^	1 [Reference]	0.72 (0.52-0.99)	0.51 (0.37-0.70)	0.42 (0.28-0.62)	NA	<.001	0.68 (0.58-0.79)	<.001
**PCa-specific mortality**								
2021 PCa Behavior Score	0-1.5	2	2.5	3	NA	NA	NA	NA
No. at risk, men (person-y)	573 (4924)	750 (7321)	796 (8062)	397 (3954)	NA	NA	2571 (24 706)	NA
No. of events	48	51	56	35	NA	NA	196	NA
Model 1, HR (95% CI)^b^	1 [Reference]	0.71 (0.47-1.09)	0.71 (0.47-1.08)	0.96 (0.60-1.55)	NA	.69	0.91 (0.71-1.18)	.50
Model 2, HR (95% CI)^c^	1 [Reference]	0.69 (0.45-1.05)	0.69 (0.45-1.07)	0.94 (0.58-1.54)	NA	.66	0.90 (0.69-1.18)	.45
2021 PCa Behavior Score Including Diet	0-1.5	1.75-2.25	2.5-3	3.25-4	NA	NA	NA	NA
No. at risk, men (person-y)	278 (2255)	719 (6734)	1071 (10 615)	503 (5103)	NA	NA	2571 (24 706)	NA
No. of events	29	49	73	45	NA	NA	196	NA
Model 1, HR (95% CI)^b^	1 [Reference]	0.62 (0.38-1.03)	0.60 (0.37-0.97)	0.79 (0.47-1.35)	NA	.62	0.93 (0.74-1.18)	.55
Model 2, HR (95% CI)^d^	1 [Reference]	0.58 (0.35-0.97)	0.59 (0.37-0.96)	0.78 (0.46-1.34)	NA	.71	0.96 (0.75-1.21)	.71

Abbreviations: CVD, cardiovascular disease; HR, hazard ratio; MEC, Multiethnic Cohort study; NA, not applicable; PCa, prostate cancer.

^a^ To maintain consistency with other studies, healthy lifestyle scores were categorized into 5 groups as in previous studies.^9,10^ For cause-specific analyses, we combined the first 2 categories of the 2021 PCa Behavior Scores and the last 2 categories of the 2021 PCa Behavior Score Including Diet because of limited participants in extreme categories.

^b^ Models were adjusted for age at diagnosis, education, race and ethnicity, family history of PCa, and total calorie intake at the follow-up questionnaire (2003-2008).

^c^ Models were adjusted for covariates in model 1 and additionally for PCa stage, grade, treatments, percentage of calories from saturated fat, whole milk intake, alcohol consumption, high-fat fish intake, tomato intake, and processed meat intake.

^d^ Models were adjusted for covariates in model 1 and additionally for PCa stage, grade, high-fat fish intake, and tomato intake.

**Figure 2.**
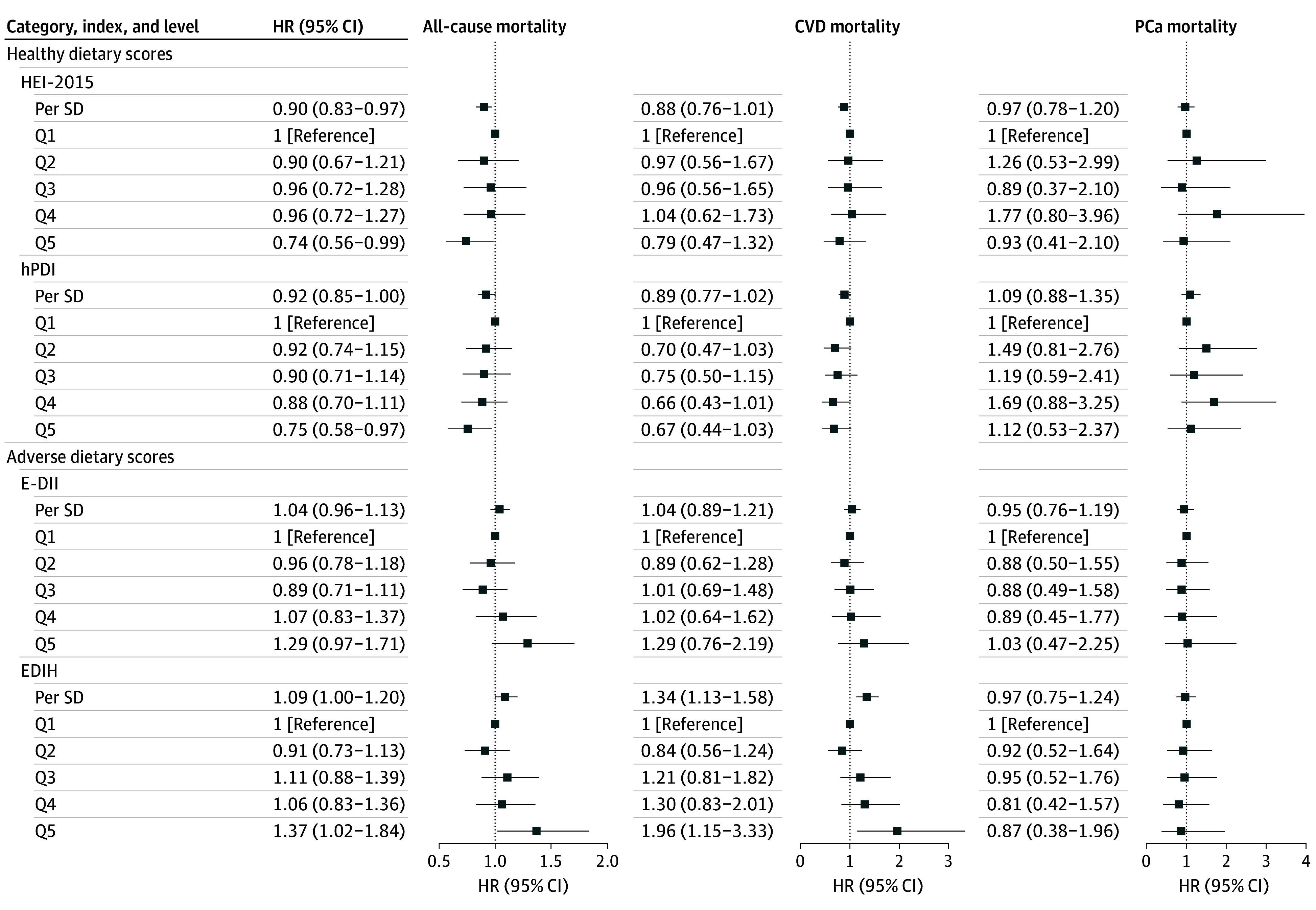
Associations Between Dietary Indices and Mortality Among Patients With Nonmetastatic Prostate Cancer (PCa) Models were adjusted for age at diagnosis, education, race and ethnicity, family history of PCa, PCa stage, grade, treatments, smoking, body mass index, physical activity, and total calorie intake at the follow-up questionnaire (2003-2008). CVD indicates cardiovascular disease; EDIH, Empirical Dietary Index for Hyperinsulinemia; E-DII, Energy-Adjusted Dietary Inflammatory Index; HEI-2015, Healthy Eating Index-2015; hPDI, Healthful Plant-Based Diet Index; HR, hazard ratio; Q, quintile.

Comparing quintile 5 (highest score) vs 1 (lowest score), greater adherence to the HEI-2015 (HR, 0.74; 95% CI, 0.56-0.99) (*P* for trend = .02) or hPDI (HR, 0.75; 95% CI, 0.58-0.97) (*P* for trend = .03) was inversely associated with all-cause mortality (Figure 2; eTable 6 in Supplement 2). A positive association was observed between EDIH and all-cause mortality (HR, 1.37; 95% CI, 1.02-1.84) (*P* for trend = .04). Participants with higher E-DII scores did not have a significantly elevated risk of all-cause mortality (HR, 1.29; 95% CI, 0.97-1.71) (*P* for trend = .25). Similarly, for CVD-related mortality, a positive association with EDIH score (HR, 1.96; 95% CI, 1.15-3.33) (*P* for trend = .01) and a nonsignificant result for HEI-2015 (HR, 0.79; 95% CI, 0.47-1.32) (*P* for trend = .27) and hPDI (HR, 0.67; 95% CI, 0.44-1.03) (*P* for trend = .07) were observed. No association was observed between the E-DII and CVD-related mortality risk. No clear trends were observed for any dietary patterns with PCa-specific mortality. Results for other dietary indices are shown in eTable 6 in Supplement 2.

When stratifying by racial and ethnic group, both the 2021 Score and 2021 Score + Diet were inversely associated with all-cause and CVD-related mortality across all groups (Table 3). The 2021 Score was significantly associated with a lower risk of PCa-specific mortality among African American participants (HR per point increase, 0.46; 95% CI, 0.24-0.88). However, no association was observed in the other racial and ethnic groups (*P* for heterogeneity = .04) (Table 3; eTable 7 in Supplement 2). Overall, the associations between dietary indices and risk of mortality varied across racial and ethnic groups (eTable 8 in Supplement 2). Inflammatory potential indices E-DII (HR, 1.82; 95% CI, 1.04-3.17) and Empirical Dietary Inflammatory Index (EDIP) (HR, 2.97; 95% CI, 1.12-7.87) were positively associated with PCa-specific mortality risk among Japanese Americans, and the Empirical Dietary Insulin Resistance Index (HR, 2.35; 95% CI, 1.02-5.42) was positively associated with PCa-specific mortality in Latino participants but not in the other racial and ethnic groups. In the sensitivity analysis stratified by prediagnostic scores, participants who maintained or improved their postdiagnostic lifestyle score tertiles showed decreased risks of all-cause and CVD-related mortality, with no clear trend for PCa-specific mortality or dietary indices (eFigure 5 in Supplement 1). In sensitivity analyses accounting for survival bias, the results remained stable (eTable 9 in Supplement 2).

**Table 3.** Associations of Healthy Lifestyle Scores and Dietary Indices With Mortality Among Men With Nonmetastatic PCa in the MEC by Race and Ethnicity (N = 2603)

Lifestyle score (per 1-point increase)^a^	African American^b^		Japanese American		Latino		White		*P* for heterogeneity^c^
	Events/No. at risk	HR (95% CI)	Events/No. at risk	HR (95% CI)	Events/No. at risk	HR (95% CI)	Events/No. at risk	HR (95% CI)	
**All-cause mortality**									
2021 PCa Behavior Score	281/484	0.80 (0.65-0.99)	376/748	0.71 (0.59-0.86)	265/568	0.64 (0.51-0.80)	331/642	0.59 (0.48-0.73)	.21
2021 PCa Behavior Score Including Diet	281/484	0.82 (0.68-0.99)	376/748	0.72 (0.60-0.85)	265/568	0.66 (0.54-0.80)	331/642	0.59 (0.49-0.71)	.10
**CVD-related mortality**									
2021 PCa Behavior Score	89/484	0.83 (0.59-1.16)	108/748	0.78 (0.57-1.08)	95/568	0.62 (0.44-0.86)	98/642	0.47 (0.33-0.67)	.01
2021 PCa Behavior Score Including Diet	89/484	0.92 (0.62-1.35)	108/748	0.81 (0.57-1.16)	95/568	0.57 (0.39-0.84)	98/642	0.39 (0.26-0.59)	.09
**PCa-specific mortality**									
2021 PCa Behavior Score	46/484	0.46 (0.24-0.88)	50/748	0.76 (0.44-1.33)	39/568	1.24 (0.65-2.37)	48/642	1.54 (0.82-2.87)	.04
2021 PCa Behavior Score Including Diet	46/484	0.70 (0.42-1.16)	50/748	0.80 (0.48-1.31)	39/568	1.27 (0.70-2.31)	48/642	1.25 (0.76-2.09)	.44

Abbreviations: CVD, cardiovascular disease; HR, hazard ratio; MEC, Multiethnic Cohort study; PCa, prostate cancer.

^a^ Models were adjusted for age at diagnosis, education, family history of PCa, total calorie intake at questionnaire follow-up 2003-2008, PCa stage, grade, treatments, and other dietary factors not included in the scores.

^b^ Results for Native Hawaiian men are not shown because of limited sample size.

^c^ The *P* value for heterogeneity was estimated using the restricted maximum likelihood method in a random-effects model via the *Q* statistic to assess the variance between racial and ethnicity groups.

## Discussion

In this prospective cohort study of men diagnosed with PCa across 5 major racial and ethnic groups in the US, we found that maintaining healthier lifestyle habits (including dietary patterns) after diagnosis was associated with a lower risk of overall mortality among those with nonmetastatic PCa. While associations varied across racial and ethnic groups, the benefits appeared to be attributed more to a reduction in CVD-related deaths rather than directly to PCa-specific mortality. We also found that proinsulinemic and proinflammatory dietary patterns assessed by E-DII and EDIH were associated with an increased risk of CVD-related deaths among patients with PCa.

All 3 lifestyle scores, the 2015 Score, 2021 Score, and 2021 Score + Diet, showed inverse trends for PCa mortality risk. Developed in the HPFS (Health Professionals Follow-Up Study), the 2015 Score was associated with a 68% lower risk of lethal PCa^20^ and a 32% reduced PCa mortality risk^9^ for men with 5 to 6 points before and after diagnosis compared with those scoring 0 to 1 point. On the other hand, the 2021 Score, which comprised factors associated with PCa recurrence, progression, or mortality, did not show an association with risk of PCa death in HPFS but was significant for the risk of PCa-specific mortality and progression in the CaPSURE (Cancer of the Prostate Strategic Urologic Research Endeavor) study when dietary factors were included in the index.^10^ The discrepancy in results across cohorts may be due to variations in timing of assessments after diagnosis and the racial and ethnic composition of the cohorts. Nonetheless, all lifestyle scores showed inverse associations with all-cause mortality in our study and regardless of prediagnostic scores, suggesting that adherence to these patterns may improve overall survival, especially the risk of CVD mortality.^28^

None of the index-based dietary patterns examined in this study were found to be associated with PCa-specific death. The observed associations varied across racial and ethnic groups, although the number of events was small in stratified analyses. Previous research on postdiagnostic dietary patterns has focused on White men. For example, the CaPSURE study found that EDIP, EDIH, Empirical Lifestyle Index for Hyperinsulinemia, and Empirical Lifestyle Insulin Resistance Index were positively associated with PCa progression; statistical power was limited in that cohort to examine PCa-specific mortality (n = 73).^12^ In our study, inflammatory and insulinemic dietary indices were significantly associated with PCa-specific mortality in Japanese American men (82% increased risk for E-DII and 197% increased risk for EDIP per SD increase) and Latinos (135% increased risk for Empirical Dietary Insulin Resistance Index), but not in other populations. In addition, while previous studies of White men have reported associations between PDIs and reduced risks of incident fatal PCa in initially healthy populations^14^ and PCa progression in men with nonmetastatic PCa,^17^ our study does not support an association of PDIs with PCa-specific mortality. These differences between studies may be due to variations in dietary habits among populations, such as cooking methods and food choices, and in dietary assessments. On the other hand, postdiagnostic healthy diets were associated with lower all-cause and CVD-related mortality in this study population. Previous MEC research similarly observed that high-quality diets were equally associated with reduced mortality among participants with and without cancer.^29^ Men with nonmetastatic PCa may equally benefit from general dietary recommendations.

### Limitations

This study has several limitations. First, the absence of information on PCa progression or recurrence constrained our ability to assess the influence of postdiagnostic lifestyle and dietary patterns across the PCa continuum. Second, the relatively small sample size of participants with PCa reduced power in analyses stratified by race and ethnicity. Additionally, further investigation is needed to confirm the outcomes associated with healthy behavior among patients with PCa in both large cohorts and randomized clinical trials, with some trials currently under way.^30,31,32,33,34^

## Conclusions

The findings of this cohort study show that healthy lifestyle and dietary patterns are associated with a lower risk of overall mortality among men with nonmetastatic PCa. However, we did not find these practices to be associated with a lower risk of PCa-specific mortality. Notably, 85% of the observed deaths during follow-up were due to causes other than PCa, highlighting the importance of managing comorbidities. Further research is warranted to assess the potential benefits of health behavior counseling for men with nonmetastatic PCa in managing comorbidities and reducing the risk of death.

## Notes

Supplement 1. **eFigure 1.** Flowchart of Inclusion and Exclusion of the Study Population **eFigure 2.** Dietary Indices Correlations Between QX1 and QX3 and at QX3 in the MEC (N = 2603) **eFigure 3.** Causes of Death After Diagnosis Among Men With Nonmetastatic Prostate Cancer in the MEC (N = 2603) **eFigure 4.** Distributions of Healthy Lifestyle Scores and Dietary Indices Among Men With Nonmetastatic Prostate Cancer in the MEC, Overall and by Race and Ethnicity (N = 2603) **eFigure 5.** Change in Healthy Lifestyle Scores and Dietary Indices from Pre and Post Diagnosis and Mortality Among Men With Nonmetastatic Prostate Cancer in the MEC (N = 2603)

Supplement 2. **eTable 1.** ICD-10 Codes and Definitions for Cause of Death **eTable 2.** Factors and Definitions of the Healthy Lifestyle Scores in the Original Studies and MEC **eTable 3.** Overview of Dietary Indices **eTable 4.** The Associations Between 2015 PCa Behavior Score and Mortality Among Men With Nonmetastatic PCa in the MEC (N = 2603) **eTable 5.** The Associations Between Selected Lifestyle Factors and Mortality in Men With Nonmetastatic Prostate Cancer in the MEC (N = 2603) **eTable 6.** The Associations Between Dietary Indices and Mortality Among Men With Nonmetastatic Prostate Cancer in the MEC (N = 2603) **eTable 7.** The Associations Between Healthy Lifestyle Score Categories and Prostate Cancer–Specific Mortality Among Men With Nonmetastatic Prostate Cancer in the MEC by Race and Ethnicity **eTable 8.** The Associations of Dietary Indices With Mortality Among Men With Nonmetastatic Prostate Cancer in the MEC by Race and Ethnicity (N = 2603) **eTable 9.** The Associations of Dietary Indices With Mortality Among Men With Nonmetastatic Prostate Cancer in the MEC, Accounting for Survival Bias (N = 5667)

Supplement 3.
Data Sharing Statement
