# Supplementary material for: Race and Ethnicity, Lifestyle, Diet, and Survival in Patients With Prostate Cancer
Source: JAMA Netw Open. 2025 Feb 26;8(2):e2460785. doi: 10.1001/jamanetworkopen.2024.60785 (PMC11866029; doi:10.1001/jamanetworkopen.2024.60785)
Supplement: Supplement 1. — eFigure 1. Flowchart of Inclusion and Exclusion of the Study Population eFigure 2. Dietary Indices Correlations Between QX1 and QX3 and at QX3 in the MEC (N = 2603) eFigure 3. Causes of Death After Diagnosis Among Men With Nonmetastatic Prostate Cancer in the MEC (N = 2603) eFigure 4. Distributions of Healthy Lifestyle Scores and Dietary Indices Among Men With Nonmetastatic Prostate Cancer in the MEC, Overall and by Race and Ethnicity (N = 2603) eFigure 5. Change in Healthy Lifestyle Scores and Dietary Indices from Pre and Post Diagnosis and Mortality Among Men With Nonmetastatic Prostate Cancer in the MEC (N = 2603) [file jamanetwopen-e2460785-s001.pdf]

Supplemental Online Content

Wang A, Van Blarigan EL, Cheng I, et al. Race and ethnicity, lifestyle, diet, and survival in patients with prostate cancer. *JAMA Netw Open*. 2025;8(2):e2460785. doi:10.1001/jamanetworkopen.2024.60785

eFigure 1. Flowchart of Inclusion and Exclusion of the Study Population

eFigure 2. Dietary Indices Correlations Between QX1 and QX3 and at QX3 in the MEC (N = 2603)

eFigure 3. Causes of Death After Diagnosis Among Men With Nonmetastatic Prostate Cancer in the MEC (N = 2603)

eFigure 4. Distributions of Healthy Lifestyle Scores and Dietary Indices Among Men With Nonmetastatic Prostate Cancer in the MEC, Overall and by Race and Ethnicity (N = 2603)

eFigure 5. Change in Healthy Lifestyle Scores and Dietary Indices from Pre and Post Diagnosis and Mortality Among Men With Nonmetastatic Prostate Cancer in the MEC (N = 2603)

This supplemental material has been provided by the authors to give readers additional information about their work.

© 2025 Wang A et al. *JAMA Network Open.*

eFigure 1. Flowchart of Inclusion and Exclusion of the Study Population

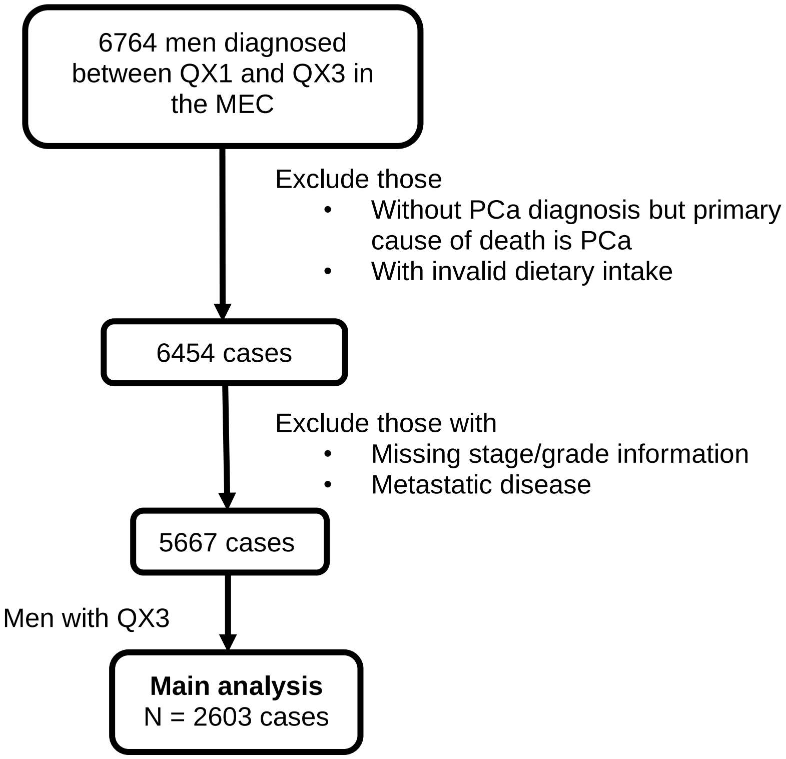

© 2025 Wang A et al. *JAMA Network Open.*

eFigure 2. Dietary Indices Correlations Between QX1 and QX3 and at QX3 in the MEC (N = 2603)

A. Correlation between dietary indices at QX1 and QX3; B.Correlation matrix of dietary indices for QX3. Square colors denote correlation magnitude, with darker colors indicating stronger correlations. Pearson correlation coefficients are displayed within each square.

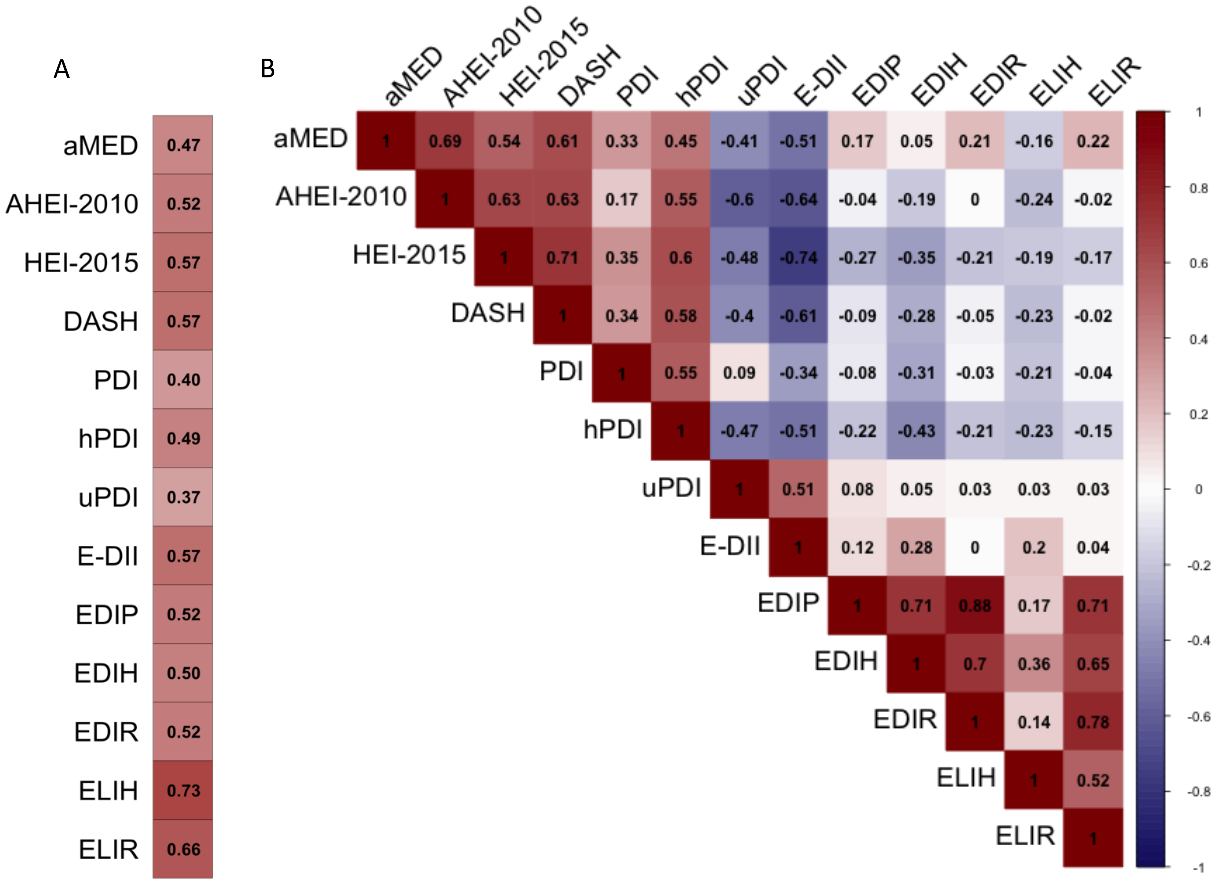

© 2025 Wang A et al. *JAMA Network Open.*

eFigure 3. Causes of Death After Diagnosis Among Men With Nonmetastatic Prostate Cancer in the MEC (N = 2603)

A. Causes of death in total population; B. Causes of death by race and ethnicity

A

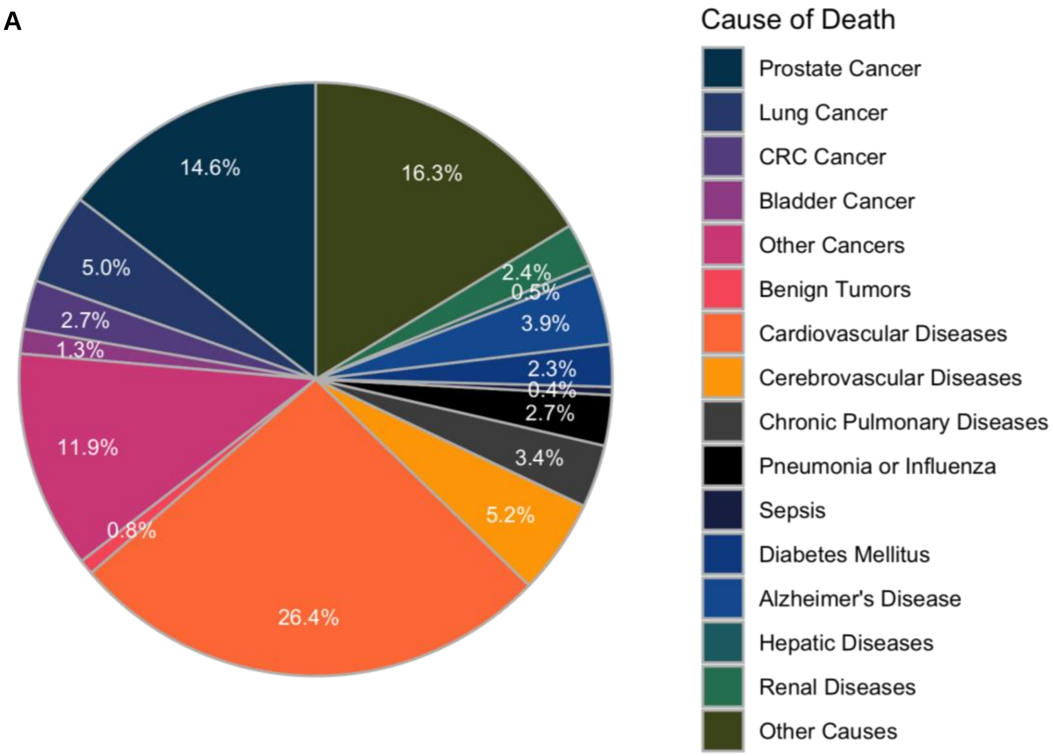

© 2025 Wang A et al. *JAMA Network Open.*

B

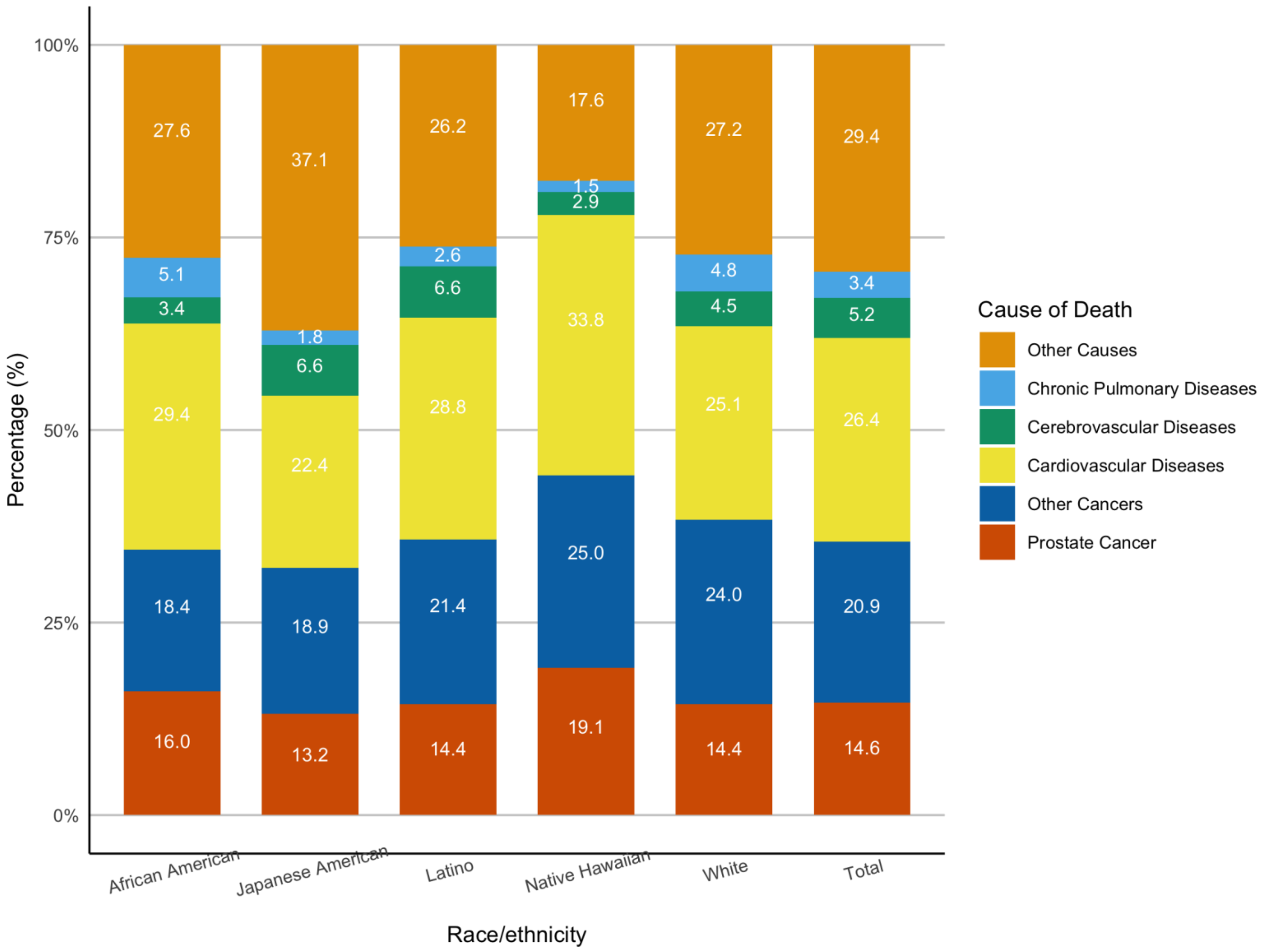

© 2025 Wang A et al. *JAMA Network Open.*

eFigure 4. Distributions of Healthy Lifestyle Scores and Dietary Indices Among Men With Nonmetastatic Prostate Cancer in the MEC, Overall and by Race and Ethnicity (N = 2603)

A. Healthy lifestyle scores. Three lifestyle scores were used to assess healthy behaviors related to prostate cancer: 2015 PCa Behavior Score (Point ranges for level 1 to level 5: 0-1, 2, 3, 4, 5-6), 2021 PCa Behavior Score (point ranges for level 1 to level 5: 0-1, 1.5, 2, 2.5, 3), and 2021 PCa Behavior Score Including Diet (point ranges: 0-1.5, 1.75-2.25, 2.5-3, 3.25-3.5, 3.75-4). B. Healthy dietary indices. Higher scores correspond to healthier dietary patterns, with quitiles based on the overall MEC population distribution. C. Adverse dietary indices. Higher scores indicate dietary patterns associated with poorer health outcomes.

© 2025 Wang A et al. *JAMA Network Open.*

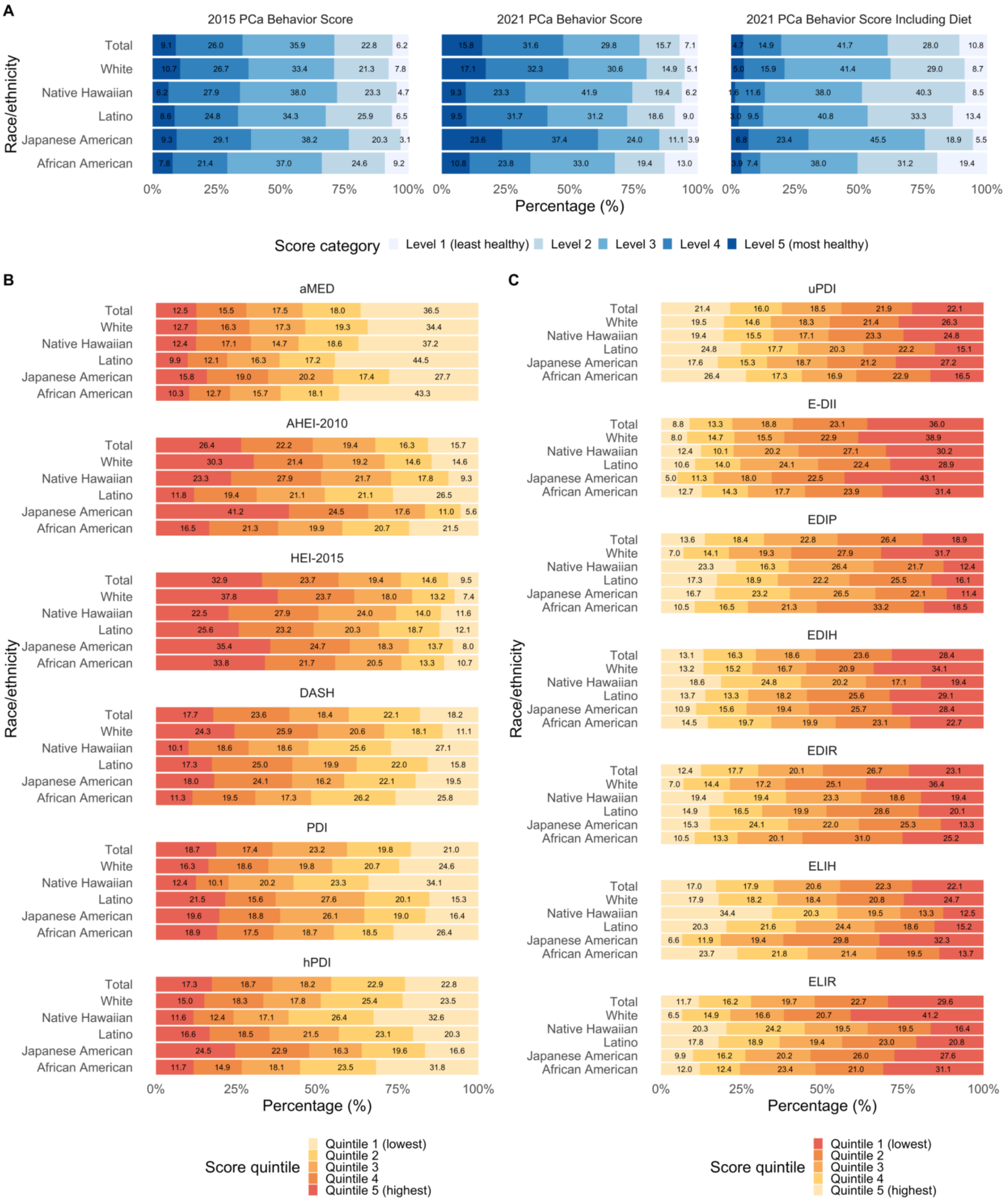

© 2025 Wang A et al. *JAMA Network Open.*

eFigure 5. Change in Healthy Lifestyle Scores and Dietary Indices From Pre and Post Diagnosis and Mortality Among Men With Nonmetastatic Prostate Cancer in the MEC (N = 2603)

A. Healthy lifestyle scores. B. Healthy dietary indices. Higher scores correspond to healthier dietary patterns. C. Adverse dietary indices. Higher scores indicate dietary patterns associated with poorer health outcomes. Tertiles were based on men included in the analysis. Models were adjusted for age at diagnosis, education, race and ethnicity, family history of prostate cancer, total calories intake at QX3, prostate cancer stage, grade, treatments, and other dietary factors not included in the scores.

A

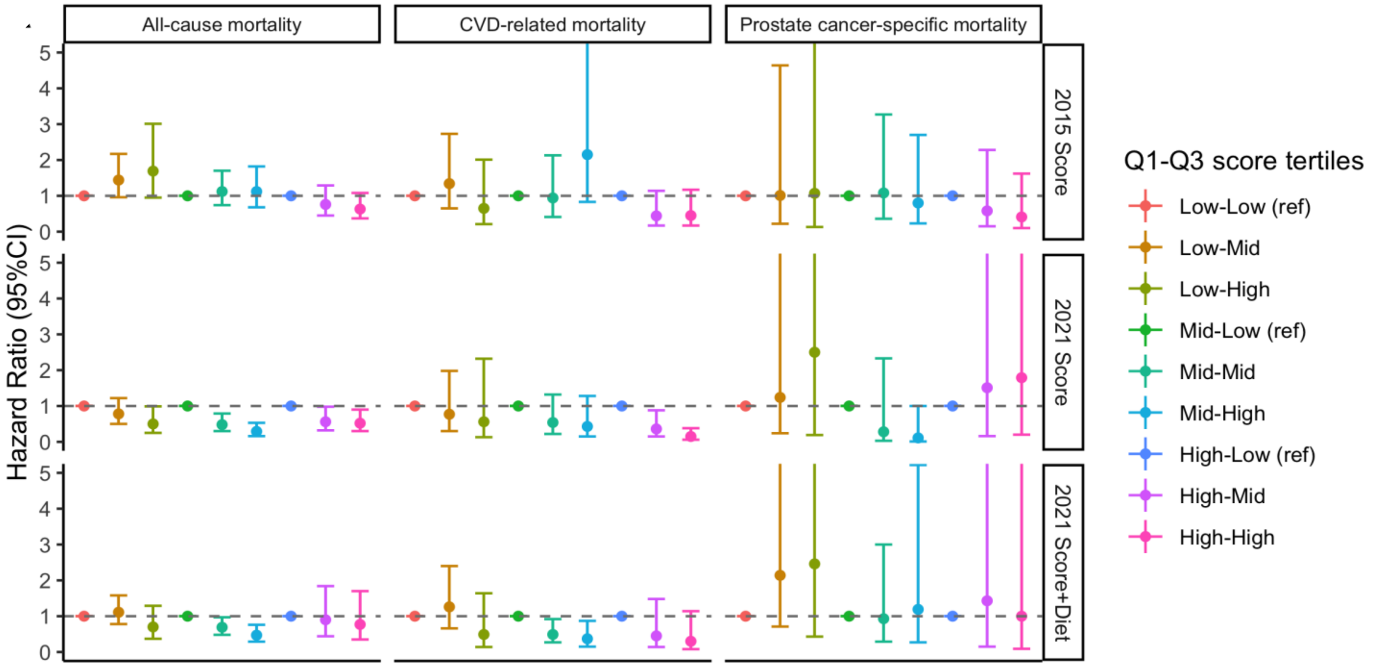

© 2025 Wang A et al. *JAMA Network Open.*

B

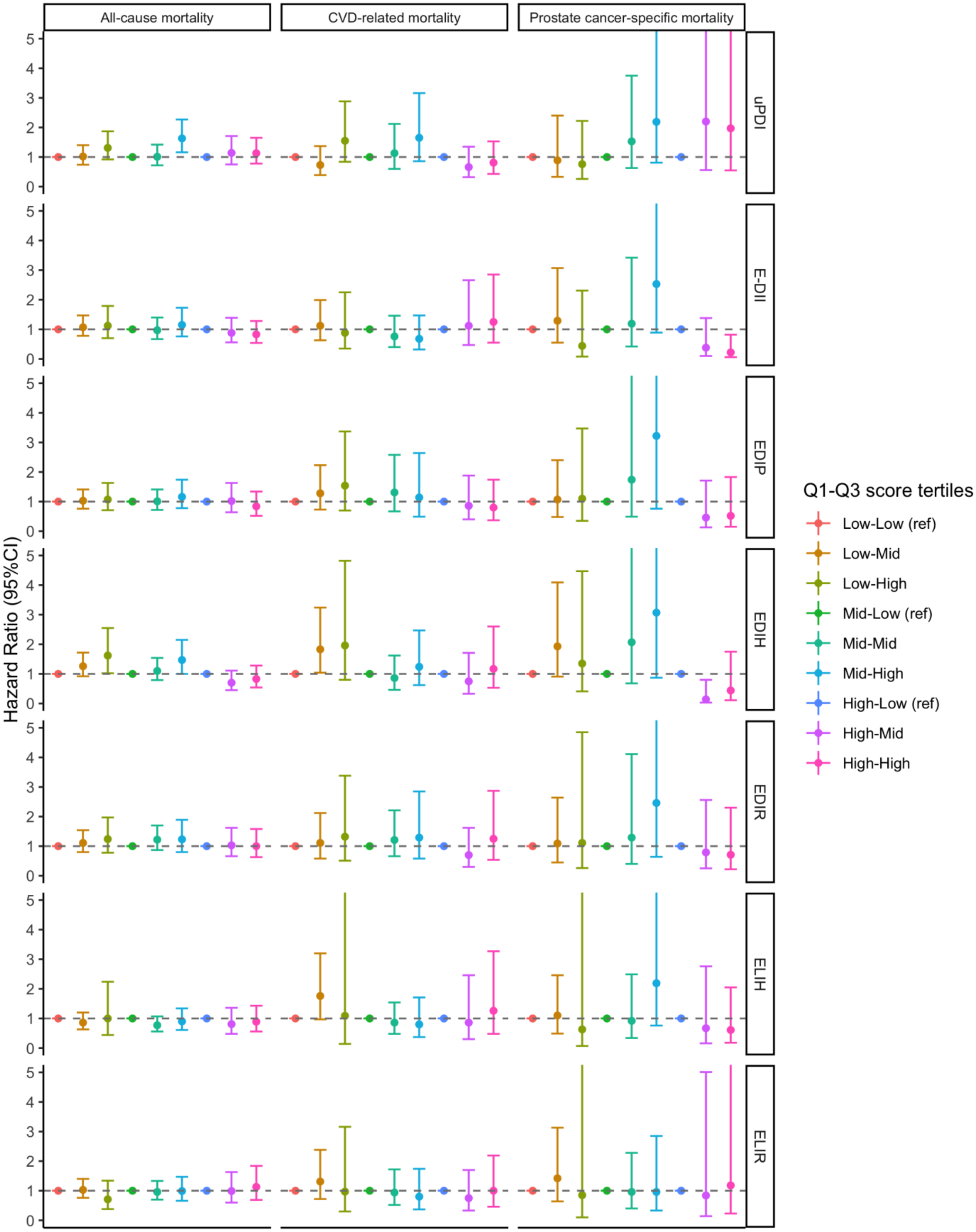

© 2025 Wang A et al. *JAMA Network Open.*

C

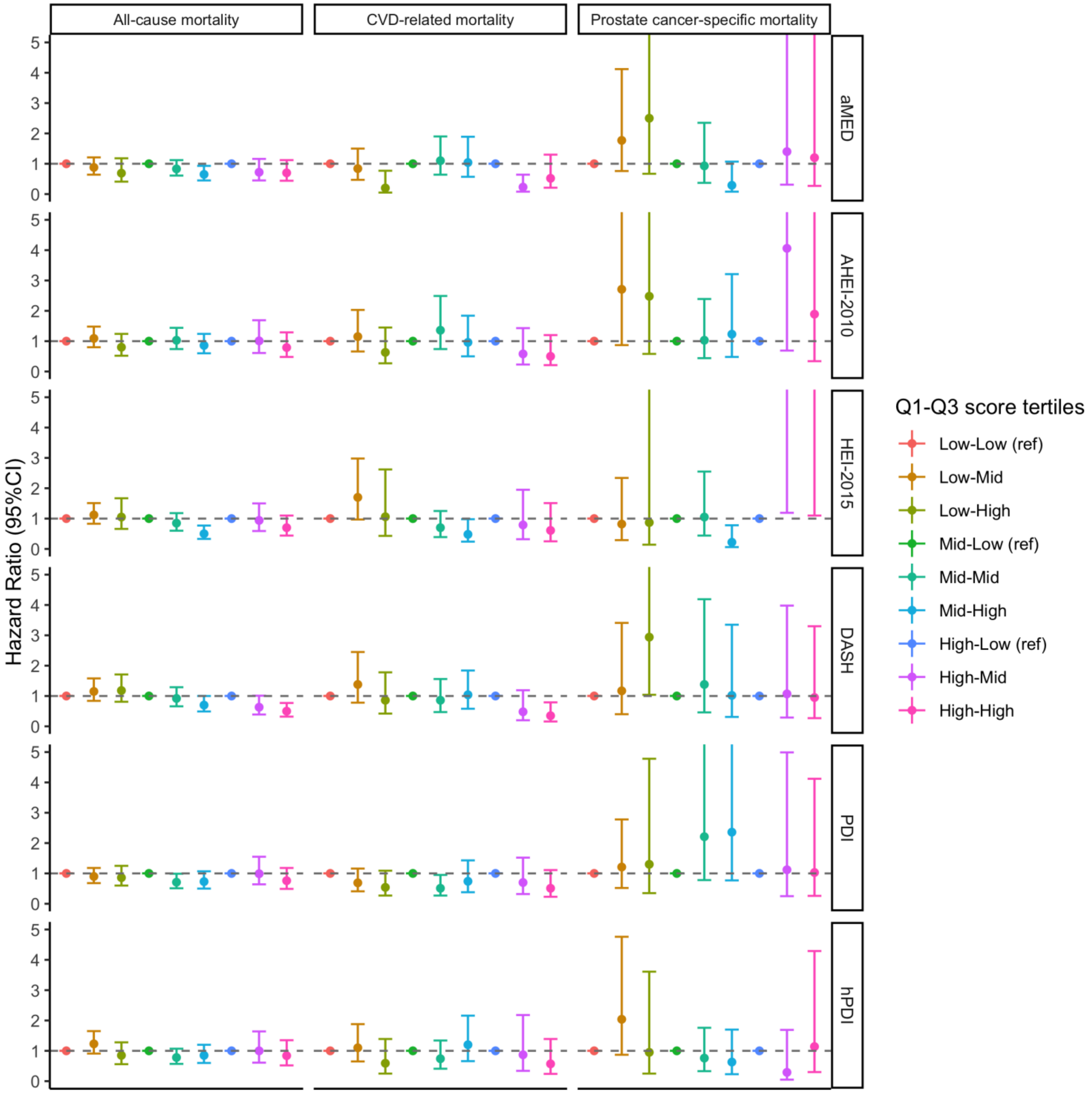

© 2025 Wang A et al. *JAMA Network Open.*
