# Supplementary material for: Race and Ethnicity, Lifestyle, Diet, and Survival in Patients With Prostate Cancer
Source: JAMA Netw Open. 2025 Feb 26;8(2):e2460785. doi: 10.1001/jamanetworkopen.2024.60785 (PMC11866029; doi:10.1001/jamanetworkopen.2024.60785)
Supplement: Supplement 2. — eTable 1. ICD-10 Codes and Definitions for Cause of Death eTable 2. Factors and Definitions of the Healthy Lifestyle Scores in the Original Studies and MEC eTable 3. Overview of Dietary Indices eTable 4. The Associations Between 2015 PCa Behavior Score and Mortality Among Men With Nonmetastatic PCa in the MEC (N = 2603) eTable 5. The Associations Between Selected Lifestyle Factors and Mortality in Men With Nonmetastatic Prostate Cancer in the MEC (N = 2603) eTable 6. The Associations Between Dietary Indices and Mortality Among Men With Nonmetastatic Prostate Cancer in the MEC (N = 2603) eTable 7. The Associations Between Healthy Lifestyle Score Categories and Prostate Cancer–Specific Mortality Among Men With Nonmetastatic Prostate Cancer in the MEC by Race and Ethnicity eTable 8. The Associations of Dietary Indices With Mortality Among Men With Nonmetastatic Prostate Cancer in the MEC by Race and Ethnicity (N = 2603) eTable 9. The Associations of Dietary Indices With Mortality Among Men With Nonmetastatic Prostate Cancer in the MEC, Accounting for Survival Bias (N = 5667) [file jamanetwopen-e2460785-s002.pdf]

Supplemental Online Content

Wang A, Van Blarigan EL, Cheng I, et al. Race and ethnicity, lifestyle, diet, and survival in patie

eTable 1. *ICD-10* Codes and Definitions for Cause of Death
eTable 2. Factors and Definitions of the Healthy Lifestyle Scores in the Original Studies
eTable 3. Overview of Dietary Indices
eTable 4. The Associations Between 2015 PCa Behavior Score and Mortality Among M
eTable 5. The Associations Between Selected Lifestyle Factors and Mortality in Men W
eTable 6. The Associations Between Dietary Indices and Mortality Among Men With Nc
eTable 7. The Associations Between Healthy Lifestyle Score Categories and Prostate C
eTable 8. The Associations of Dietary Indices With Mortalities Among Men With Nonme
eTable 9. The Associations of Dietary Indices With Mortality Among Men With Nonmeta

This supplemental material has been provided by the authors to give readers additional informa

© 2025 Wang A et al. *JAMA Network Open* .

nts with prostate cancer. *JAMA Netw Open* . 2025;8(2):e2460785. doi:10.1001/jamanetworkope

:ancer-Specific Mortality Among Men With Nonmetastatic Prostate Cancer in the MEC b

astatic Prostate Cancer in the MEC, Accounting for Survival Bias (N = 5667)

© 2025 Wang A et al. *JAMA Network Open* .

n.2024.60785

›y Race and Ethnicity

© 2025 Wang A et al. *JAMA Network Open* .

eTable 1. ***ICD-10*** **Codes and Definitions for Cause of Death**

Category	Cause of death	ICD-10 code
All	All	Any
Cancer	Prostate	C61
	Other cancer	Any cancer, not C61
	Lung/bronchus	C34
	Colorectal	C18, C26.0
		C19-C20
	Urinary bladder	C67
	Other	Any cancer, not C61, C34, C18, C26.0, C19-20, or C67
Non-cancer	Non-cancer	
	Benign tumors	D00-D49
		D50-D89
	Cardiovascular Diseases	I00-I02
		I05-I09
		I10-I15
		I20-I25
		I26-I28
		I30-I52
		I70-I79
		I80-I89
		I95-I99
	Cerebrovascular Diseases	I60-I69
	Chronic pulmonary diseases	J40-J42
		J43
		J44
		J45-J46
		J47
	Pneumonia or influenza	J09-J18
	Sepsis	A40-A41
	Diabetes mellitus	E10-E14
	Alzheimer's	G30
	Hepatic	K70
		K73-K74
	Renal	N00-N07
		N17-N19
		N25
		N26
		N27
	Accidents	V01-V99
		W00-X59
		Y85-Y86
	Suicide or self-harm	U03, X60-X84
		Y87
	Other	Any other non-cancer

© 2025 Wang A et al. *JAMA Network Open.*

**Definition**
Any
Prostate cancer
Any cancer, not prostate cancer
Lung and Bronchus
Colon excluding Rectum
Rectum and Rectosigmoid Junction
Urinary bladder
Any cancer, not prostate cancer, colorectal, lung, bronchus, or urinary bladder
Any non-cancer death
In situ and benign neoplasms
Diseases of the blood and blood-forming organs and certain disorders involving the immune mechanism
Acute rheumatic fever
Chronic rheumatic heart diseases
Hypertensive diseases
Ischemic heart diseases
Pulmonary heart disease and diseases of pulmonary circulation
Other forms of heart disease
Diseases of arteries, arterioles and capillaries
Diseases of veins, lymphatic vessels and lymph nodes, not elsewhere classified
Other and unspecified disorders of the circulatory system
Cerebrovascular diseases
Bronchitis
Emphysema
Other chronic obstructive pulmonary disease
Asthma or Status asthmaticus
Bronchiectasis
Influenza and pneumonia
Sepsis
Diabetes mellitus
Alzheimer's disease
Alcoholic liver disease
Chronic hepatitis and fibrosis and cirrhosis of liver
Glomerular diseases
Acute kidney failure and chronic kidney disease
Disorders resulting from impaired renal tubular function
Unspecified contracted kidney
Small kidney of unknown cause
Transportation accidents
Other accidental, external causes
Sequelae of transport accidents or other accidents
Intentional self-harm
Sequelae of intentional self-harm, assault and events of undetermined intent
Any other non-cancer

© 2025 Wang A et al. *JAMA Network Open.*

eTable 2. Factors and Definitions of the Healthy Lifestyle Scores in th

Score (range)	2021 PCa Behavior Score (0-3)		2021 PCa Behavior Sco
**Lifestyle Factor**	**Definition in HPFS**	**Definition in MEC**	**Definition in HPFS**
**BMI**	18.5-<24.9 kg/m^2 = 1 point;<br>25-<30 kg/m^2 = 0.5 point;<br>else 0 point		18.5-<24.9 kg/<br>25-<30 kg/m^2<br>else 0
**Physical activity**	≥ 18 MET-h/wk vigorous activity= 1 point;<br>9-<18 MET-h/wk vigorous activity= 0.5 point;<br>else 0 point	≥ 18 MET-h/wk moderate and vigorous activity= 1 point;<br>9-<18 MET-h/wk moderate and vigorous activity= 0.5 point;<br>else 0 point	≥ 18 MET-h/wk vigorous activity= 1 point;<br>9-<18 MET-h/wk vigorous activity= 0.5 point;<br>else 0 point
**Smoking status**	Never smokers or quit ≥ 10 years age = 1 point;<br>Quit<10 years age = 0.5 point;<br>current 0 point		Never smokers or quit ≥<br>Quit<10 years a<br>current
**Tomatoes intake**	-		-
**Fatty fish intake**	-		-

© 2025 Wang A et al. *JAMA Network Open* .

**Processed meat intake**	-	< 2 servings/wk of<br>beef or pork hot dogs (1),<br>bacon (2 slices),<br>salami, bologna, or other processed meat sandwiches (1),<br>OR other processed meats (2 oz)<br>= 1 point; else 0 point
**Percentage of calories from saturated Fat**	-	<10% calories from satura[illegible]<br>poir[illegible]
**Whole milk intake**	-	≤ 4 servings/wk = 1 [illegible]
**Alcohol consumption**	-	3-14 servings/wk of wine [illegible]

[a] All score components were converted to uniform units to ensure comparability across var[illegible]

© 2025 Wang A et al. *JAMA Network Open* .

e Original Studies and MEC

re Including Diet (0-4)	2015 PCa Behavior Score (0-6)	
Definition in MEC	Definition in HPFS/NHS	Definition in MEC
m^2 = 1 point;<br>= 0.5 point;<br>point	< 30 kg/m^2 = 1 point, else 0 point	
≥ 18 MET-h/wk moderate and vigorous activity= 1 point;<br>9-<18 MET-h/wk moderate and vigorous activity= 0.5 point;<br>else 0 point	≥ 3 h/wk vigorous activity OR ≥ 7 h/wk brisk walking = 1 point; else 0 point	≥ 3 h/wk strenuous sports or vigorous work=1 point; else 0 point
10 years age = 1 point;<br>ge = 0.5 point;<br>) point	Never smokers or quite ≥ 10 years age = 1 point, else 0 point	
	≥ 7 servings/wk of raw tomatoes (1),<br>tomato juice (small glass),<br>tomato sauce (1/2 cup),<br>salsa (1/4 cup),<br>OR pizza (2 slices)<br>= 1 point; else 0 point	≥3.5 cup-equivalents/wk of tomato intake (including raw tomatoes, tomato juice, ingredients in salsa, catsup, and mixed dish items such as tomato soup, pizza, pasta, salad, tacos, etc)<br>= 1 point; else 0 point
	≥1 serving/wk of mackerel, salmon, sardines, bluefish, OR swordfish = 1 point; else 0 point	≥1 serving/wk of cooked fishe or shell fish high in n-3 fatty acids (e.g., tuna, salmon steak, trout, etc.)<br>= 1 point; else 0 point

© 2025 Wang A et al. *JAMA Network Open* .

< 2 servings/wk of cooked lean meat from frankfurters, sausages, and luncheon meats = 1point; else 0 point	< 3 servings/wk of beef or pork hot dogs (1), bacon (2 slices), salami, bologna, or other processed meat sandwiches (1), OR other processed meats (2 oz) = 1 point; else 0 point	< 3 servings/wk of cooked lean meat from frankfurters, sausages, and luncheon meats = 1point; else 0 point
ated fat= 1 point; else 0 nt	-	
point; else 0 point	-	
= 1 point; else 0 point	-	

ious studies

© 2025 Wang A et al. *JAMA Network Open* .

eTable 3. Overview of Dietary Indices

Category		Index[a]
**Dietary Patterns Methods Project Indices**	**Healthy dietary indices**	Healthy Eating Index-2015 (HEI-2015)
		Alternate Healthy Eating Index-2010 (AHEI-2010)
		Alternate Mediterranean Diet Score (aMED)
		Dietary Approaches to Stop Hypertension (DASH) Index
**Plant-Based Diet Indices**		Plant-Based Diet Index (PDI)
		Healthful Plant-Based Diet Index (hPDI)
	**Adverse dietary indices**	Unhealthful Plant-Based Diet Index (uPDI)
**Empirical Indices for Inflammatory and Insulinemic Potential**		Energy-Adjusted Dietary Inflammatory Index (E-DII)
		Empirical Dietary Inflammatory Index (EDIP)
		Empirical Dietary Insulinemic Index (EDIH)
		Empirical Lifestyle Insulinemic Index (ELIH)
		Empirical Dietary Insulin Resistance Index (EDIR)
		Empirical Lifestyle Insulin Resistance Index (ELIR)

[a]Lower scores indicate lesser adherence to the dietary pattern, while higher scores indicate greater adhe

© 2025 Wang A et al. *JAMA Network Open* .

Description
To evaluate adherence to the 2015-2020 Dietary Guidelines for Americans.
To evaluate diet quality in relation to chronic disease prevention.
To evaluate adherence to Mediterranean dietary patterns.
To evaluate diet effectiveness in managing hypertension.
To evaluate overall plant food consumption.
To evaluate plant food consumption, focusing on healthy plant foods.
To evaluate plant food consumption, focusing on less healthy plant foods.
To evaluate dietary inflammatory potential, with total energy intake adjusted.
To evaluate the inflammatory potential of diet.
To evaluate dietary patterns related to hyperinsulinemia.
To evaluate dietary patterns and behavioral factors (BMI and physical activity) related to hyperinsulinemia.
To evaluate dietary patterns related to insulin resistance.
To evaluate dietary patterns and behavioral factors (BMI and physical activity) related to insulin resistance.

erence.

© 2025 Wang A et al. *JAMA Network Open* .

eTable 4. The Associations Between 2015 PCa Behavior Score and Mortal

		Healthy Lifestyle	
**2015 PCa Behavior Score**		**0-1**	**2**
	**No./person-year at risk**	161/1481	587/5460
**All-cause Mortality**			
	**Events**	92	339
	**Model 1 HR (95%CI)[a]**	1 [Reference]	0.94 (0.74,1.20)
	**Model 2 HR (95%CI)[b]**	1 [Reference]	1.00 (0.78,1.28)
**CVD-Related Mortality**			
	**Events**	29	118
	**Model 1 HR (95%CI)[a]**	1 [Reference]	1.06 (0.69,1.65)
	**Model 2 HR (95%CI)[b]**	1 [Reference]	1.15 (0.73,1.79)
**Prostate Cancer-Specific Mortality**			
	**Events**	14	42
	**Model 1 HR (95%CI)[a]**	1 [Reference]	0.87 (0.46,1.63)
	**Model 2 HR (95%CI)[b]**	1 [Reference]	0.89 (0.46,1.69)

[a] Models were adjusted for age at diagnosis, education, race and ethnicity, family history of pros

[b] Models were adjusted for covariates in Model 1, and additionally for prostate cancer stage, gra

© 2025 Wang A et al. *JAMA Network Open* .

ity Among Men With Nonmetastatic PCa in the MEC (N = 2603)

Scores Categories				Per Point Increase	p-value
3	4	5-6	P for trend		
926/8669	672/6729	234/2459		2580/24798	
507	305	84		1327	
0.94 (0.74,1.18)	0.76 (0.60,0.98)	0.61 (0.45,0.84)	<0.001	0.90 (0.85,0.95)	<0.001
1.02 (0.80,1.29)	0.83 (0.65,1.07)	0.72 (0.52,1.00)	0.003	0.92 (0.87,0.97)	0.004
152	87	30		416	
0.95 (0.62,1.46)	0.74 (0.47,1.16)	0.69 (0.40,1.20)	0.009	0.87 (0.79,0.96)	0.005
1.08 (0.70,1.68)	0.85 (0.53,1.34)	0.90 (0.51,1.60)	0.11	0.91 (0.83,1.01)	0.08
73	54	13		196	
0.87 (0.48,1.61)	0.93 (0.50,1.73)	0.67 (0.30,1.47)	0.59	0.96 (0.84,1.11)	0.61
0.94 (0.50,1.76)	0.92 (0.48,1.75)	0.67 (0.30,1.53)	0.54	0.96 (0.83,1.11)	0.57

tate cancer, total calories intake at QX3;

de, treatments, percentage of calories from saturated fat, whole milk intake, and wine consumption.

© 2025 Wang A et al. *JAMA Network Open* .

eTable 5. The Associations Between Selected Lifestyle Factors and Mortali

		All-cause Mortality	
	No. at risk	Events	Model 1 HR (95%CI)[a]
**Smoking status**			
Never	760	326	1 [Reference]
Former: quit>10y	1095	578	1.22 (1.06,1.40)
Former: quit≤10y	311	173	1.49 (1.24,1.80)
Current	148	98	2.03 (1.61,2.56)
**BMI category**			
Normal	902	498	1 [Reference]
Underweight	17	11	1.12 (0.61,2.05)
Overweight	1019	474	0.90 (0.79,1.03)
Obese	376	192	1.23 (1.03,1.48)
**Moderate and vigorous physical activity**			
< 9 MET-h/wk	358	252	1 [Reference]
9-<18 MET-h/wk	329	200	0.74 (0.61,0.89)
≥18 MET-h/wk	1627	723	0.52 (0.45,0.60)
**Tomatoes intake**			
< 1 serving/day	1816	938	1 [Reference]
≥1 serving/day	498	237	0.94 (0.80,1.10)
**Fish high in n-3 fatty acids intake**			
< 1 serving/wk	592	324	1 [Reference]
≥1 serving/wk	1722	851	0.92 (0.80,1.06)
**Processed meat intake**			
≥2 servings/wk	1407	712	1 [Reference]
≤2 servings/wk	907	463	0.96 (0.84,1.09)
**Percentage of calories from saturated fat**			
≥10%	1026	551	1 [Reference]
<10%	1288	624	0.85 (0.75,0.96)
**Whole milk intake**			
>4 servings/wk	1124	594	1 [Reference]
≤4 servings/wk	1190	581	1.05 (0.93,1.19)
**Wine consumption**			
<3 or >14 servings/wk	2086	1085	1 [Reference]
3-14 servings/wk	228	90	0.64 (0.51,0.80)
**Total coffee intake[c]**			
None	422	213	1 [Reference]
≤1 cup/day	1304	678	1.00 (0.85,1.17)
>1 to 2 cups/day	182	82	0.83 (0.64,1.07)
>2 cups/day	406	202	1.01 (0.83,1.23)
**Long-term (>1 yr) supplemental selenium intake[c]**			
None	1442	772	1 [Reference]
0-<140 mcg/day	749	353	0.86 (0.75,0.97)
≥140 mcg/day	123	50	0.70 (0.53,0.94)
**Cruciferous vegetables intake[c]**			
Quintile 1 (≤6.28 g/day)	458	254	1 [Reference]
Quintile 2 (6.29-11.44 g/day)	458	229	0.87 (0.72,1.04)
Quintile 3 (11.45-17.37 g/day)	463	218	0.77 (0.64,0.93)
Quintile 4 (17.38-28.79 g/day)	471	233	0.84 (0.70,1.02)
Quintile 5 (>28.79 g/day)	464	241	0.86 (0.71,1.03)
**High-fat dairy intake[c]**			
Quintile 1 (≤4.41 g/day)	474	237	1 [Reference]
Quintile 2 (4.42-12.84 g/day)	461	187	0.80 (0.66,0.97)

© 2025 Wang A et al. *JAMA Network Open* .

**Quintile 3 (12.85-27.00 g/day)**	466	222	0.91 (0.76,1.10)
**Quintile 4 (27.01-67.51 g/day)**	460	239	1.03 (0.85,1.24)
**Quintile 5 (>67.51 g/day)**	453	290	1.31 (1.09,1.58)

[a] Models were adjusted for age at diagnosis, education, race and ethnicity, family history of prost

[b] Models were adjusted for covariates in Model 1, and additionally for prostate cancer stage, grac

[c] Factors that not included in the healthy behavior scores, but have shown evidence of impacting

© 2025 Wang A et al. *JAMA Network Open* .

ity in Men With Nonmetastatic Prostate Cancer in the MEC (N = 2603)

/	CVD-related Mortality			P
Model 2 HR (95%CI)[b]	Events	Model 1 HR (95%CI)	Model 2 HR (95%CI)	Events
1 [Reference]	103	1 [Reference]	1 [Reference]	58
1.22 (1.06,1.40)	183	1.22 (0.95,1.56)	1.19 (0.93,1.53)	85
1.39 (1.14,1.68)	57	1.52 (1.09,2.12)	1.30 (0.93,1.83)	16
1.89 (1.49,2.39)	30	2.05 (1.35,3.12)	1.76 (1.15,2.69)	12
1 [Reference]	153	1 [Reference]	1 [Reference]	70
1.23 (0.67,2.26)	0	-	-	2
0.87 (0.76,0.99)	152	0.99 (0.78,1.26)	0.95 (0.75,1.21)	68
1.07 (0.89,1.29)	68	1.52 (1.11,2.07)	1.37 (1.00,1.88)	31
1 [Reference]	85	1 [Reference]	1 [Reference]	31
0.76 (0.63,0.93)	68	0.75 (0.54,1.04)	0.79 (0.57,1.10)	20
0.55 (0.47,0.64)	220	0.49 (0.38,0.64)	0.53 (0.40,0.69)	120
1 [Reference]	294	1 [Reference]	1 [Reference]	137
1.01 (0.86,1.19)	79	1.06 (0.80,1.39)	1.13 (0.85,1.49)	34
1 [Reference]	108	1 [Reference]	1 [Reference]	51
0.94 (0.81,1.08)	265	0.95 (0.74,1.21)	0.94 (0.74,1.21)	120
1 [Reference]	213	1 [Reference]	1 [Reference]	93
1.01 (0.89,1.15)	160	1.03 (0.82,1.29)	1.14 (0.91,1.44)	78
1 [Reference]	183	1 [Reference]	1 [Reference]	80
0.95 (0.84,1.08)	190	0.78 (0.63,0.97)	0.87 (0.69,1.09)	91
1 [Reference]	184	1 [Reference]	1 [Reference]	89
1.05 (0.93,1.19)	189	1.09 (0.88,1.36)	1.12 (0.90,1.41)	82
1 [Reference]	343	1 [Reference]	1 [Reference]	158
0.71 (0.57,0.89)	30	0.70 (0.48,1.02)	0.82 (0.56,1.22)	13
1 [Reference]	66	1 [Reference]	1 [Reference]	39
0.96 (0.82,1.13)	221	1.07 (0.81,1.42)	0.99 (0.75,1.32)	95
0.80 (0.61,1.04)	29	0.93 (0.59,1.45)	0.88 (0.56,1.39)	13
0.94 (0.76,1.15)	57	0.97 (0.67,1.39)	0.80 (0.55,1.17)	24
1 [Reference]	257	1 [Reference]	1 [Reference]	110
0.91 (0.80,1.04)	103	0.79 (0.63,1.00)	0.85 (0.67,1.08)	48
0.76 (0.57,1.02)	13	0.56 (0.32,0.99)	0.66 (0.37,1.16)	13
1 [Reference]	88	1 [Reference]	1 [Reference]	33
0.93 (0.77,1.12)	72	0.81 (0.59,1.11)	0.85 (0.61,1.17)	39
0.88 (0.72,1.06)	67	0.68 (0.49,0.95)	0.79 (0.56,1.11)	26
0.96 (0.79,1.16)	70	0.76 (0.54,1.05)	0.85 (0.61,1.20)	32
1.02 (0.85,1.24)	76	0.82 (0.59,1.13)	0.99 (0.70,1.38)	41
1 [Reference]	78	1 [Reference]	1 [Reference]	31
0.81 (0.66,0.98)	51	0.64 (0.45,0.91)	0.60 (0.41,0.86)	22

© 2025 Wang A et al. *JAMA Network Open* .

0.89 (0.73,1.08)	75	0.91 (0.66,1.26)	0.81 (0.57,1.14)	39
1.02 (0.84,1.25)	86	1.11 (0.80,1.53)	1.00 (0.71,1.42)	34
1.21 (0.98,1.49)	83	1.16 (0.83,1.61)	0.92 (0.63,1.33)	45

ate cancer, total calories intake at QX3;
le, treatments and other lifestyle factors in the table;
prostate cancer progression or mortality in prior studies.

© 2025 Wang A et al. *JAMA Network Open* .

Prostate cancer-specific mortality	
Model 1 HR (95%CI)[a]	Model 2 HR (95%CI)[b]
1 [Reference]	1 [Reference]
0.99 (0.70,1.39)	1.00 (0.71,1.42)
0.71 (0.40,1.24)	0.70 (0.40,1.25)
1.16 (0.62,2.19)	1.10 (0.57,2.11)
1 [Reference]	1 [Reference]
1.48 (0.36,6.12)	1.63 (0.38,6.92)
0.82 (0.58,1.17)	0.80 (0.56,1.15)
1.09 (0.69,1.72)	0.97 (0.60,1.56)
1 [Reference]	1 [Reference]
0.58 (0.33,1.02)	0.63 (0.36,1.13)
0.71 (0.48,1.08)	0.75 (0.49,1.13)
1 [Reference]	1 [Reference]
0.89 (0.59,1.35)	0.89 (0.59,1.35)
1 [Reference]	1 [Reference]
0.78 (0.54,1.11)	0.81 (0.56,1.17)
1 [Reference]	1 [Reference]
1.39 (1.00,1.93)	1.40 (0.99,1.98)
1 [Reference]	1 [Reference]
0.94 (0.69,1.30)	0.91 (0.65,1.27)
1 [Reference]	1 [Reference]
0.95 (0.69,1.31)	0.99 (0.71,1.37)
1 [Reference]	1 [Reference]
0.64 (0.36,1.15)	0.72 (0.40,1.30)
1 [Reference]	1 [Reference]
0.79 (0.54,1.15)	0.80 (0.54,1.18)
0.76 (0.40,1.43)	0.81 (0.42,1.54)
0.64 (0.38,1.08)	0.77 (0.45,1.32)
1 [Reference]	1 [Reference]
0.82 (0.58,1.16)	0.84 (0.59,1.19)
1.35 (0.75,2.43)	1.35 (0.74,2.47)
1 [Reference]	1 [Reference]
1.12 (0.70,1.79)	1.17 (0.72,1.89)
0.73 (0.43,1.23)	0.79 (0.46,1.35)
0.92 (0.56,1.53)	1.00 (0.59,1.68)
1.17 (0.72,1.87)	1.24 (0.75,2.05)
1 [Reference]	1 [Reference]
0.70 (0.40,1.21)	0.79 (0.45,1.37)

© 2025 Wang A et al. *JAMA Network Open* .

1.26 (0.78,2.04)	1.43 (0.87,2.38)
1.10 (0.66,1.82)	1.22 (0.71,2.09)
1.54 (0.94,2.52)	1.61 (0.93,2.79)

© 2025 Wang A et al. *JAMA Network Open* .

eTable 6. The Associations Between Dietary Indices and Mortality Among M

			Dietary	
			**Quintile 1**	**Quintile 2**
**All-cause Mortality**				
**Healthy dietary indices**	**aMED**	**No./person-year at risk**	951/8909	469/4421
		**Events**	517	246
		**Model 1 HR (95%CI)[a]**	1 [Reference]	0.87 (0.74,1.03)
		**Model 2 HR (95%CI)[b]**	1 [Reference]	0.82 (0.66,1.03)
	**AHEI-2010**	**No./person-year at risk**	408/3758	425/3984
		**Events**	225	226
		**Model 1 HR (95%CI)[a]**	1 [Reference]	0.91 (0.74,1.11)
		**Model 2 HR (95%CI)[b]**	1 [Reference]	0.94 (0.72,1.22)
	**HEI-2015**	**No./person-year at risk**	246/2168	380/3492
		**Events**	147	208
		**Model 1 HR (95%CI)[a]**	1 [Reference]	0.82 (0.65,1.03)
		**Model 2 HR (95%CI)[b]**	1 [Reference]	0.90 (0.67,1.21)
	**DASH**	**No./person-year at risk**	473/4417	574/5288
		**Events**	249	307
		**Model 1 HR (95%CI)[a]**	1 [Reference]	1.02 (0.85,1.22)
		**Model 2 HR (95%CI)[b]**	1 [Reference]	1.09 (0.87,1.38)
	**PDI**	**No./person-year at risk**	546/5135	515/4841
		**Events**	292	271
		**Model 1 HR (95%CI)[a]**	1 [Reference]	0.94 (0.78,1.12)
		**Model 2 HR (95%CI)[b]**	1 [Reference]	1.01 (0.80,1.27)
	**hPDI**	**No./person-year at risk**	594/5457	596/5667
		**Events**	328	313
		**Model 1 HR (95%CI)[a]**	1 [Reference]	0.91 (0.77,1.08)
		**Model 2 HR (95%CI)[b]**	1 [Reference]	0.92 (0.74,1.15)
**Adverse dietary indices**	**uPDI**	**No./person-year at risk**	576/5719	570/5598
		**Events**		289
		**Model 1 HR (95%CI)[a]**	1 [Reference]	0.96 (0.80,1.14)
		**Model 2 HR (95%CI)[b]**	1 [Reference]	0.84 (0.66,1.06)
	**E-DII**	**No./person-year at risk**	938/9130	601/5797
		**Events**	486	295
		**Model 1 HR (95%CI)[a]**	1 [Reference]	1.01 (0.86,1.17)
		**Model 2 HR (95%CI)[b]**	1 [Reference]	0.96 (0.78,1.18)
	**EDIP**	**No./person-year at risk**	492/4800	687/6508
		**Events**	250	359
		**Model 1 HR (95%CI)[a]**	1 [Reference]	1.01 (0.85,1.21)
		**Model 2 HR (95%CI)[b]**	1 [Reference]	0.94 (0.74,1.18)
	**EDIH**	**No./person-year at risk**	740/7216	614/6018
		**Events**	378	296
		**Model 1 HR (95%CI)[a]**	1 [Reference]	0.95 (0.80,1.11)
		**Model 2 HR (95%CI)[b]**	1 [Reference]	0.91 (0.73,1.13)
	**EDIR**	**No./person-year at risk**	601/5812	696/6694
		**Events**	314	345
		**Model 1 HR (95%CI)[a]**	1 [Reference]	0.95 (0.81,1.12)
		**Model 2 HR (95%CI)[b]**	1 [Reference]	0.83 (0.67,1.03)
	**ELIH**	**No./person-year at risk**	568/5375	572/5531
		**Events**	305	276
		**Model 1 HR (95%CI)[a]**	1 [Reference]	0.98 (0.82,1.16)

© 2025 Wang A et al. *JAMA Network Open* .

		**Model 2 HR (95%CI)[d]**	1 [Reference]	1.00 (0.80,1.25)
	**ELIR**	**No./person-year at risk**	760/7206	583/5676
		**Events**	399	291
		**Model 1 HR (95%CI)[a]**	1 [Reference]	0.97 (0.82,1.14)
		**Model 2 HR (95%CI)[d]**	1 [Reference]	0.96 (0.78,1.17)
**CVD-Related Mortality**				
**Healthy dietary indices**	**aMED**	**No./person-year at risk**	951/8909	469/4421
		**Events**	171	67
		**Model 1 HR (95%CI)[a]**	1 [Reference]	0.77 (0.57,1.04)
		**Model 2 HR (95%CI)[b]**	1 [Reference]	0.68 (0.45,1.03)
	**AHEI-2010**	**No./person-year at risk**	408/3758	425/3984
		**Events**	83	68
		**Model 1 HR (95%CI)[a]**	1 [Reference]	0.77 (0.54,1.09)
		**Model 2 HR (95%CI)[b]**	1 [Reference]	0.91 (0.56,1.46)
	**HEI-2015**	**No./person-year at risk**	246/2168	380/3492
		**Events**	45	68
		**Model 1 HR (95%CI)[a]**	1 [Reference]	0.91 (0.61,1.37)
		**Model 2 HR (95%CI)[b]**	1 [Reference]	0.97 (0.56,1.67)
	**DASH**	**No./person-year at risk**	473/4417	574/5288
		**Events**	75	101
		**Model 1 HR (95%CI)[a]**	1 [Reference]	1.17 (0.85,1.61)
		**Model 2 HR (95%CI)[b]**	1 [Reference]	1.15 (0.75,1.74)
	**PDI**	**No./person-year at risk**	546/5135	515/4841
		**Events**	102	78
		**Model 1 HR (95%CI)[a]**	1 [Reference]	0.78 (0.57,1.09)
		**Model 2 HR (95%CI)[b]**	1 [Reference]	0.70 (0.46,1.07)
	**hPDI**	**No./person-year at risk**	594/5457	596/5667
		**Events**	116	89
		**Model 1 HR (95%CI)[a]**	1 [Reference]	0.74 (0.55,0.99)
		**Model 2 HR (95%CI)[b]**	1 [Reference]	0.70 (0.47,1.03)
**Adverse dietary indices**	**uPDI**	**No./person-year at risk**	576/5719	570/5598
		**Events**	94	87
		**Model 1 HR (95%CI)[a]**	1 [Reference]	0.85 (0.62,1.16)
		**Model 2 HR (95%CI)[b]**	1 [Reference]	0.67 (0.44,1.03)
	**E-DII**	**No./person-year at risk**	938/9130	601/5797
		**Events**	150	97
		**Model 1 HR (95%CI)[a]**	1 [Reference]	1.07 (0.82,1.40)
		**Model 2 HR (95%CI)[b]**	1 [Reference]	0.89 (0.62,1.28)
	**EDIP**	**No./person-year at risk**	492/4800	687/6508
		**Events**	77	115
		**Model 1 HR (95%CI)[a]**	1 [Reference]	1.02 (0.75,1.39)
		**Model 2 HR (95%CI)[b]**	1 [Reference]	0.96 (0.63,1.44)
	**EDIH**	**No./person-year at risk**	740/7216	614/6018
		**Events**	116	91
		**Model 1 HR (95%CI)[a]**	1 [Reference]	0.97 (0.73,1.30)
		**Model 2 HR (95%CI)[b]**	1 [Reference]	0.84 (0.56,1.24)
	**EDIR**	**No./person-year at risk**	601/5812	696/6694
		**Events**	96	115
		**Model 1 HR (95%CI)[a]**	1 [Reference]	1.04 (0.78,1.39)
		**Model 2 HR (95%CI)[b]**	1 [Reference]	0.84 (0.58,1.22)
	**ELIH**	**No./person-year at risk**	568/5375	572/5531

© 2025 Wang A et al. *JAMA Network Open* .

		Events	79	92
		Model 1 HR (95%CI)[a]	1 [Reference]	1.31 (0.95,1.80)
		Model 2 HR (95%CI)[d]	1 [Reference]	1.37 (0.91,2.06)
	ELIR	No./person-year at risk	760/7206	583/5676
		Events	109	101
		Model 1 HR (95%CI)[a]	1 [Reference]	1.26 (0.94,1.69)
		Model 2 HR (95%CI)[d]	1 [Reference]	1.14 (0.79,1.64)
**Prostate Cancer-Specific Mortality**				
Healthy dietary indices	aMED	No./person-year at risk	951/8909	469/4421
		Events	67	39
		Model 1 HR (95%CI)[a]	1 [Reference]	1.12 (0.72,1.75)
		Model 2 HR (95%CI)[b]	1 [Reference]	1.21 (0.66,2.20)
	AHEI-2010	No./person-year at risk	408/3758	425/3984
		Events	21	34
		Model 1 HR (95%CI)[a]	1 [Reference]	1.27 (0.70,2.32)
		Model 2 HR (95%CI)[b]	1 [Reference]	1.34 (0.59,3.03)
	HEI-2015	No./person-year at risk	246/2168	380/3492
		Events	16	29
		Model 1 HR (95%CI)[a]	1 [Reference]	0.88 (0.45,1.71)
		Model 2 HR (95%CI)[b]	1 [Reference]	1.26 (0.53,2.99)
	DASH	No./person-year at risk	473/4417	574/5288
		Events	29	42
		Model 1 HR (95%CI)[a]	1 [Reference]	1.41 (0.84,2.38)
		Model 2 HR (95%CI)[b]	1 [Reference]	0.90 (0.45,1.79)
	PDI	No./person-year at risk	546/5135	515/4841
		Events	35	42
		Model 1 HR (95%CI)[a]	1 [Reference]	1.41 (0.87,2.30)
		Model 2 HR (95%CI)[b]	1 [Reference]	2.19 (1.14,4.22)
	hPDI	No./person-year at risk	594/5457	596/5667
		Events	41	52
		Model 1 HR (95%CI)[a]	1 [Reference]	1.43 (0.92,2.24)
		Model 2 HR (95%CI)[b]	1 [Reference]	1.49 (0.81,2.76)
Adverse dietary indices	uPDI	No./person-year at risk	576/5719	570/5598
		Events	42	44
		Model 1 HR (95%CI)[a]	1 [Reference]	1.01 (0.64,1.59)
		Model 2 HR (95%CI)[b]	1 [Reference]	0.92 (0.50,1.68)
	E-DII	No./person-year at risk	938/9130	601/5797
		Events	82	35
		Model 1 HR (95%CI)[a]	1 [Reference]	0.64 (0.41,0.97)
		Model 2 HR (95%CI)[b]	1 [Reference]	0.88 (0.50,1.55)
	EDIP	No./person-year at risk	492/4800	687/6508
		Events	43	49
		Model 1 HR (95%CI)[a]	1 [Reference]	0.82 (0.53,1.29)
		Model 2 HR (95%CI)[b]	1 [Reference]	0.82 (0.43,1.59)
	EDIH	No./person-year at risk	740/7216	614/6018
		Events	57	48
		Model 1 HR (95%CI)[a]	1 [Reference]	0.95 (0.63,1.44)
		Model 2 HR (95%CI)[b]	1 [Reference]	0.92 (0.52,1.64)
	EDIR	No./person-year at risk	601/5812	696/6694
		Events	55	49
		Model 1 HR (95%CI)[a]	1 [Reference]	0.78 (0.52,1.19)

© 2025 Wang A et al. *JAMA Network Open* .

	**Model 2 HR (95%CI)[b]**	1 [Reference]	0.69 (0.38,1.25)
**ELIH**	**No./person-year at risk**	568/5375	572/5531
	**Events**	46	40
	**Model 1 HR (95%CI)[a]**	1 [Reference]	0.94 (0.60,1.47)
	**Model 2 HR (95%CI)[d]**	1 [Reference]	1.11 (0.62,1.96)
**ELIR**	**No./person-year at risk**	760/7206	583/5676
	**Events**	62	37
	**Model 1 HR (95%CI)[a]**	1 [Reference]	0.81 (0.53,1.24)
	**Model 2 HR (95%CI)[d]**	1 [Reference]	1.06 (0.62,1.82)

[a] Models were adjusted for age at diagnosis, education, race and ethnicity, family history of prosta

[b] Models were adjusted for covariates in Model 1, and additionally for prostate cancer stage, grad

[c] Models were adjusted for covariates in Model 2, and additionally for lifestyle scores at QX1;

[d] Models were adjusted for covariates in Model 1, and additionally for smoking.

© 2025 Wang A et al. *JAMA Network Open* .

Men With Nonmetastatic Prostate Cancer in the MEC (N = 2603)

Indices Quintiles					
Quintile 3	Quintile 4	Quintile 5	P for trend	Per SD Increase	p-value
455/4334	403/3996	325/3320		2603/24979	
233	199	151		1346	
0.85 (0.71,1.01)	0.71 (0.59,0.86)	0.69 (0.56,0.86)	<0.001	0.85 (0.80,0.91)	<0.001
0.87 (0.69,1.11)	0.77 (0.60,0.98)	0.70 (0.53,0.93)	0.007	0.88 (0.80,0.96)	0.003
506/4740	577/5616	687/6882		2603/24979	
268	290	337		1346	
0.85 (0.70,1.03)	0.78 (0.65,0.95)	0.74 (0.61,0.90)	0.001	0.90 (0.84,0.95)	0.001
1.08 (0.84,1.39)	0.91 (0.71,1.18)	0.81 (0.63,1.05)	0.07	0.92 (0.85,1.00)	0.05
504/4775	617/5960	856/8584		2603/24979	
270	316	405		1346	
0.78 (0.63,0.97)	0.73 (0.59,0.91)	0.62 (0.51,0.77)	<0.001	0.86 (0.81,0.91)	<0.001
0.96 (0.72,1.28)	0.96 (0.72,1.27)	0.74 (0.56,0.99)	0.02	0.90 (0.83,0.97)	0.008
480/4612	614/6027	462/4636		2603/24979	
253	317	220		1346	
0.87 (0.72,1.06)	0.83 (0.69,1.00)	0.75 (0.61,0.92)	0.001	0.89 (0.84,0.95)	<0.001
0.98 (0.76,1.27)	1.01 (0.80,1.29)	0.83 (0.64,1.08)	0.13	0.95 (0.88,1.03)	0.24
603/5799	452/4456	487/4748		2603/24979	
317	221	245		1346	
0.86 (0.72,1.02)	0.78 (0.65,0.95)	0.78 (0.65,0.93)	0.002	0.90 (0.85,0.95)	<0.001
0.83 (0.66,1.04)	0.78 (0.61,1.00)	0.82 (0.64,1.06)	0.03	0.90 (0.83,0.97)	0.008
474/4633	488/4723	451/4499		2603/24979	
238	254	213		1346	
0.82 (0.69,0.98)	0.81 (0.68,0.97)	0.70 (0.58,0.84)	<0.001	0.89 (0.84,0.95)	<0.001
0.90 (0.71,1.14)	0.88 (0.70,1.11)	0.75 (0.58,0.97)	0.03	0.92 (0.85,1.00)	0.05
482/4650	417/3900	558/5113		2603/24979	
242	232	304		1346	
1.00 (0.83,1.20)	1.11 (0.92,1.34)	1.24 (1.04,1.48)	0.006	1.10 (1.04,1.17)	0.001
0.94 (0.75,1.19)	1.00 (0.78,1.28)	1.26 (1.01,1.58)	0.02	1.10 (1.02,1.18)	0.02
489/4794	345/3212	230/2046		2603/24979	
244	189	132		1346	
0.98 (0.83,1.15)	1.22 (1.02,1.47)	1.38 (1.11,1.70)	0.005	1.09 (1.03,1.16)	0.005
0.89 (0.71,1.11)	1.07 (0.83,1.37)	1.29 (0.97,1.71)	0.25	1.04 (0.96,1.13)	0.34
593/5833	478/4433	353/3405		2603/24979	
284	267	186		1346	
0.94 (0.78,1.13)	1.11 (0.91,1.35)	1.19 (0.93,1.52)	0.18	1.04 (0.97,1.12)	0.31
0.98 (0.77,1.25)	1.09 (0.85,1.40)	1.07 (0.78,1.49)	0.48	1.02 (0.92,1.12)	0.73
484/4527	423/4076	342/3144		2603/24979	
253	224	195		1346	
1.17 (0.98,1.39)	1.18 (0.98,1.42)	1.42 (1.14,1.77)	0.001	1.11 (1.04,1.19)	0.002
1.11 (0.88,1.39)	1.06 (0.83,1.36)	1.37 (1.02,1.84)	0.04	1.09 (0.99,1.20)	0.07
522/5020	461/4365	323/3089		2603/24979	
275	242	170		1346	
1.07 (0.90,1.28)	0.99 (0.81,1.20)	1.12 (0.87,1.44)	0.46	1.01 (0.94,1.09)	0.7
1.08 (0.86,1.35)	0.95 (0.74,1.22)	1.04 (0.76,1.42)	0.78	0.99 (0.91,1.09)	0.89
529/5197	459/4428	437/4150		2565/24682	
267	231	238		1317	
1.05 (0.88,1.25)	1.11 (0.92,1.34)	1.27 (1.05,1.54)	0.007	1.09 (1.03,1.16)	0.003

© 2025 Wang A et al. *JAMA Network Open* .

1.00 (0.81,1.24)	1.07 (0.85,1.34)	1.11 (0.88,1.41)	0.33	1.06 (0.98,1.14)	0.16
506/4802	415/4081	301/2917		2565/24682	
272	200	155		1317	
1.12 (0.94,1.32)	0.98 (0.80,1.19)	1.21 (0.94,1.56)	0.22	1.05 (0.97,1.13)	0.22
1.13 (0.91,1.40)	0.94 (0.74,1.20)	1.10 (0.81,1.50)	0.65	1.03 (0.94,1.13)	0.55
455/4334	403/3996	325/3320		2603/24979	
78	66	44		426	
0.93 (0.68,1.26)	0.80 (0.57,1.11)	0.73 (0.50,1.07)	0.09	0.88 (0.78,0.99)	0.03
0.94 (0.62,1.43)	0.76 (0.49,1.18)	0.81 (0.50,1.33)	0.3	0.91 (0.77,1.06)	0.23
506/4740	577/5616	687/6882		2603/24979	
88	89	98		426	
0.75 (0.54,1.05)	0.70 (0.50,0.97)	0.66 (0.48,0.92)	0.02	0.89 (0.79,0.99)	0.03
1.04 (0.66,1.64)	1.00 (0.64,1.56)	0.69 (0.44,1.10)	0.13	0.90 (0.78,1.05)	0.18
504/4775	617/5960	856/8584		2603/24979	
86	106	121		426	
0.87 (0.58,1.29)	0.83 (0.57,1.22)	0.66 (0.45,0.96)	0.008	0.85 (0.77,0.95)	0.003
0.96 (0.56,1.65)	1.04 (0.62,1.73)	0.79 (0.47,1.32)	0.27	0.88 (0.76,1.01)	0.07
480/4612	614/6027	462/4636		2603/24979	
85	102	63		426	
1.01 (0.71,1.42)	0.91 (0.66,1.27)	0.78 (0.54,1.12)	0.06	0.89 (0.80,0.99)	0.04
0.97 (0.60,1.55)	1.14 (0.74,1.75)	0.77 (0.47,1.26)	0.35	0.94 (0.81,1.08)	0.36
603/5799	452/4456	487/4748		2603/24979	
98	73	75		426	
0.82 (0.61,1.11)	0.78 (0.56,1.07)	0.71 (0.52,0.98)	0.05	0.88 (0.79,0.97)	0.01
0.63 (0.42,0.94)	0.62 (0.41,0.96)	0.67 (0.44,1.03)	0.05	0.83 (0.72,0.96)	0.01
474/4633	488/4723	451/4499		2603/24979	
71	86	64		426	
0.70 (0.51,0.97)	0.80 (0.59,1.08)	0.59 (0.42,0.82)	0.005	0.86 (0.77,0.95)	0.004
0.75 (0.50,1.15)	0.66 (0.43,1.01)	0.67 (0.44,1.03)	0.07	0.89 (0.77,1.02)	0.1
482/4650	417/3900	558/5113		2603/24979	
71	74	100		426	
0.90 (0.65,1.25)	1.00 (0.72,1.39)	1.18 (0.87,1.60)	0.2	1.10 (0.99,1.22)	0.08
0.93 (0.62,1.39)	0.89 (0.58,1.38)	1.11 (0.75,1.64)	0.46	1.07 (0.93,1.22)	0.35
489/4794	345/3212	230/2046		2603/24979	
79	57	43		426	
1.02 (0.77,1.37)	1.19 (0.85,1.66)	1.39 (0.96,2.03)	0.11	1.09 (0.98,1.21)	0.12
1.01 (0.69,1.48)	1.02 (0.64,1.62)	1.29 (0.76,2.19)	0.5	1.04 (0.89,1.21)	0.62
593/5833	478/4433	353/3405		2603/24979	
94	83	57		426	
0.95 (0.68,1.31)	1.17 (0.82,1.66)	1.35 (0.87,2.10)	0.21	1.19 (1.03,1.36)	0.02
0.89 (0.58,1.38)	1.13 (0.72,1.77)	0.90 (0.49,1.67)	1	1.12 (0.92,1.36)	0.25
484/4527	423/4076	342/3144		2603/24979	
81	73	65		426	
1.28 (0.94,1.74)	1.40 (1.01,1.94)	2.05 (1.40,2.99)	<0.001	1.29 (1.15,1.45)	<0.001
1.21 (0.81,1.82)	1.30 (0.83,2.01)	1.96 (1.15,3.33)	0.01	1.34 (1.13,1.58)	0.001
522/5020	461/4365	323/3089		2603/24979	
73	89	53		426	
0.95 (0.68,1.33)	1.27 (0.90,1.79)	1.53 (0.97,2.41)	0.07	1.18 (1.03,1.36)	0.02
0.81 (0.53,1.25)	1.08 (0.69,1.68)	1.02 (0.57,1.82)	0.88	1.10 (0.92,1.33)	0.3
529/5197	459/4428	437/4150		2565/24682	

© 2025 Wang A et al. *JAMA Network Open* .

86	74	83		414	
1.31 (0.94,1.83)	1.44 (1.02,2.05)	1.88 (1.34,2.65)	<0.001	1.26 (1.14,1.39)	<0.001
1.19 (0.79,1.80)	1.35 (0.88,2.08)	1.70 (1.09,2.65)	0.03	1.19 (1.04,1.37)	0.01
506/4802	415/4081	301/2917		2565/24682	
86	68	50		414	
1.46 (1.07,1.99)	1.38 (0.97,1.96)	1.99 (1.29,3.07)	0.002	1.30 (1.14,1.49)	<0.001
1.40 (0.94,2.06)	1.29 (0.83,2.00)	1.43 (0.81,2.52)	0.15	1.19 (1.00,1.42)	0.05
455/4334	403/3996	325/3320		2603/24979	
37	27	27		197	
1.30 (0.83,2.03)	0.89 (0.54,1.48)	1.10 (0.64,1.88)	0.85	0.99 (0.83,1.17)	0.88
1.50 (0.81,2.79)	1.04 (0.53,2.06)	1.05 (0.50,2.21)	0.78	1.02 (0.80,1.29)	0.87
506/4740	577/5616	687/6882		2603/24979	
41	45	56		197	
1.54 (0.88,2.70)	1.40 (0.80,2.46)	1.52 (0.87,2.64)	0.18	1.05 (0.89,1.23)	0.57
2.16 (1.02,4.56)	1.33 (0.60,2.94)	1.79 (0.84,3.79)	0.22	1.05 (0.85,1.30)	0.65
504/4775	617/5960	856/8584		2603/24979	
38	48	66		197	
1.04 (0.57,1.93)	1.08 (0.60,1.96)	1.06 (0.60,1.88)	0.62	1.06 (0.91,1.24)	0.45
0.89 (0.37,2.10)	1.77 (0.80,3.96)	0.93 (0.41,2.10)	0.74	0.97 (0.78,1.20)	0.77
480/4612	614/6027	462/4636		2603/24979	
42	44	40		197	
1.49 (0.87,2.57)	1.31 (0.77,2.23)	1.65 (0.96,2.84)	0.13	1.10 (0.94,1.29)	0.24
1.14 (0.57,2.31)	1.09 (0.55,2.13)	1.17 (0.58,2.38)	0.54	1.05 (0.84,1.31)	0.65
603/5799	452/4456	487/4748		2603/24979	
49	27	44		197	
1.16 (0.71,1.87)	0.98 (0.57,1.67)	1.39 (0.85,2.27)	0.46	1.04 (0.89,1.21)	0.66
1.41 (0.70,2.87)	0.98 (0.45,2.14)	2.03 (1.01,4.08)	0.29	1.11 (0.89,1.37)	0.36
474/4633	488/4723	451/4499		2603/24979	
32	39	33		197	
1.02 (0.62,1.69)	1.20 (0.74,1.95)	1.12 (0.67,1.88)	0.9	1.02 (0.88,1.19)	0.78
1.19 (0.59,2.41)	1.69 (0.88,3.25)	1.12 (0.53,2.37)	0.64	1.09 (0.88,1.35)	0.43
482/4650	417/3900	558/5113		2603/24979	
39	37	35		197	
1.12 (0.70,1.79)	1.24 (0.77,2.01)	0.89 (0.54,1.47)	0.96	1.01 (0.86,1.18)	0.92
1.06 (0.56,1.99)	1.13 (0.59,2.15)	1.01 (0.53,1.93)	0.8	1.01 (0.82,1.24)	0.92
489/4794	345/3212	230/2046		2603/24979	
39	25	16		197	
0.85 (0.57,1.29)	0.88 (0.54,1.42)	0.78 (0.43,1.42)	0.38	0.91 (0.78,1.07)	0.26
0.88 (0.49,1.58)	0.89 (0.45,1.77)	1.03 (0.47,2.25)	0.84	0.95 (0.76,1.19)	0.64
593/5833	478/4433	353/3405		2603/24979	
41	36	28		197	
0.79 (0.50,1.25)	0.78 (0.47,1.28)	0.82 (0.44,1.54)	0.41	0.88 (0.74,1.04)	0.14
0.93 (0.48,1.80)	1.23 (0.62,2.44)	1.26 (0.54,2.92)	0.43	1.09 (0.83,1.43)	0.54
484/4527	423/4076	342/3144		2603/24979	
33	29	30		197	
0.93 (0.59,1.46)	0.79 (0.48,1.30)	0.87 (0.48,1.55)	0.5	0.88 (0.73,1.05)	0.14
0.95 (0.52,1.76)	0.81 (0.42,1.57)	0.87 (0.38,1.96)	0.66	0.97 (0.75,1.24)	0.79
522/5020	461/4365	323/3089		2603/24979	
36	35	22		197	
0.78 (0.50,1.23)	0.72 (0.44,1.17)	0.50 (0.26,0.99)	0.04	0.84 (0.71,0.99)	0.04

© 2025 Wang A et al. *JAMA Network Open* .

1.10 (0.60,2.00)	0.98 (0.50,1.90)	0.85 (0.37,1.91)	0.88	1.01 (0.79,1.30)	0.92
529/5197	459/4428	437/4150		2565/24682	
30	41	37		194	
0.80 (0.49,1.30)	1.04 (0.64,1.68)	1.01 (0.62,1.65)	0.85	1.05 (0.90,1.23)	0.53
0.95 (0.52,1.74)	1.05 (0.57,1.96)	0.74 (0.38,1.43)	0.37	1.03 (0.84,1.26)	0.81
506/4802	415/4081	301/2917		2565/24682	
40	23	32		194	
0.99 (0.64,1.54)	0.63 (0.36,1.08)	1.16 (0.63,2.17)	0.94	0.93 (0.77,1.13)	0.48
1.28 (0.73,2.26)	0.54 (0.26,1.13)	1.20 (0.56,2.56)	0.96	1.01 (0.79,1.29)	0.96

ıte cancer, total calories intake at QX3;

e, treatments, BMI, smoking, and physical activity;

© 2025 Wang A et al. *JAMA Network Open* .

eTable 7. The Associations Between Healthy Lifestyle Score Categories and Prostate Ca MEC by Race and Ethnicity

	African American		Japanese American	
	Events/No. at risk	HR (95%CI)	Events/No. at risk	HR (95%CI)
**2021 PCa Behavior Score**				
**0-1.5**	15/94	1 [Reference]	2/41	1 [Reference]
**2**	13/151	0.82 (0.30,2.25)	11/141	1.26 (0.25,6.48)
**2.5**	11/184	0.30 (0.10,0.93)	24/340	1.07 (0.23,5.02)
**3**	7/55	0.63 (0.17,2.33)	13/226	0.81 (0.17,3.96)
**P for trend**		0.09		0.17
**2021 PCa Behavior Score Including Diet**				
**0-1.5**	18/150	1 [Reference]	10/111	1 [Reference]
**1.75-2.25**	12/153	0.49 (0.17,1.39)	12/177	0.61 (0.24,1.57)
**2.5-3**	6/110	0.21 (0.06,0.76)	17/276	0.48 (0.19,1.21)
**3.25-4**	6/50	0.60 (0.16,2.31)	11/174	0.51 (0.19,1.34)
**P for trend**		0.09		0.42

[a] Models were adjusted for age at diagnosis, education, family history of prostate cancer, total calories intake at QX

[b] Results for Native Hawaiians were not shown due to limited sample size.

© 2025 Wang A et al. *JAMA Network Open* .

ncer-Specific Mortality Among Men With Nonmetastatic Prostate Cancer in the

Latino		White	
Events/No. at risk	HR (95%CI)	Events/No. at risk	HR (95%CI)
8/76	1 [Reference]	3/56	1 [Reference]
8/189	0.19 (0.05,0.68)	12/186	0.66 (0.17,2.59)
13/232	0.25 (0.08,0.78)	18/266	0.79 (0.22,2.90)
10/71	1.40 (0.47,4.16)	15/134	0.97 (0.25,3.74)
	0.23		0.12
10/153	1 [Reference]	6/126	1 [Reference]
10/173	0.60 (0.20,1.80)	11/193	1.17 (0.39,3.50)
12/176	0.96 (0.34,2.73)	18/204	1.62 (0.58,4.56)
6/53	2.30 (0.71,7.49)	12/108	2.17 (0.72,6.54)
	0.34		0.58

‹3, prostate cancer stage, grade, treatments, and other dietary factors not included in the scores;

© 2025 Wang A et al. *JAMA Network Open* .

eTable 8. The Associations of Dietary Indices With Mortality Among Men With Nonm

		African American		Japanese American	
		Events/No. at risk	HR (95%CI)	Events/No. at risk	HR (95%CI)
**All-Cause Mortality**					
**Dietary indices (per SD increase)[a]**					
**Healthy deitary indices**	**aMED**	293/497	0.90 (0.72,1.12)	380/754	0.89 (0.75,1.07)
	**AHEI-2010**	293/497	0.95 (0.77,1.17)	380/754	0.96 (0.81,1.14)
	**HEI-2015**	293/497	0.93 (0.76,1.14)	380/754	0.89 (0.76,1.04)
	**DASH**	293/497	1.00 (0.81,1.22)	380/754	0.87 (0.75,1.02)
	**PDI**	293/497	0.83 (0.68,1.00)	380/754	0.88 (0.75,1.04)
	**hPDI**	293/497	0.94 (0.77,1.14)	380/754	0.89 (0.76,1.04)
**Adverse dietary indices**	**uPDI**	293/497	1.03 (0.86,1.24)	380/754	1.12 (0.97,1.31)
	**E-DII**	293/497	0.97 (0.78,1.20)	380/754	1.01 (0.85,1.19)
	**EDIP**	293/497	0.88 (0.68,1.12)	380/754	1.15 (0.94,1.41)
	**EDIH**	293/497	0.83 (0.65,1.07)	380/754	1.14 (0.95,1.38)
	**EDIR**	293/497	0.92 (0.74,1.14)	380/754	1.03 (0.84,1.26)
	**ELIH**	279/482	0.87 (0.72,1.04)	374/746	1.11 (0.92,1.32)
	**ELIR**	279/482	0.95 (0.76,1.18)	374/746	0.96 (0.76,1.20)
**CVD-Related Mortality**					
**Dietary indices (per SD increase)[a]**					
**Healthy deitary indices**	**aMED**	96/497	0.98 (0.62,1.54)	110/754	1.50 (1.06,2.12)
	**AHEI-2010**	96/497	1.11 (0.72,1.71)	110/754	1.14 (0.84,1.56)
	**HEI-2015**	96/497	0.97 (0.66,1.44)	110/754	1.01 (0.75,1.36)
	**DASH**	96/497	1.00 (0.68,1.47)	110/754	1.06 (0.81,1.40)
	**PDI**	96/497	0.71 (0.50,1.01)	110/754	1.12 (0.82,1.53)
	**hPDI**	96/497	0.92 (0.64,1.32)	110/754	1.05 (0.79,1.39)
**Adverse dietary indices**	**uPDI**	96/497	0.74 (0.51,1.07)	110/754	1.05 (0.81,1.37)
	**E-DII**	96/497	0.69 (0.45,1.07)	110/754	0.71 (0.52,0.98)
	**EDIP**	96/497	1.25 (0.66,2.37)	110/754	0.89 (0.60,1.32)
	**EDIH**	96/497	1.45 (0.88,2.38)	110/754	1.02 (0.71,1.45)
	**EDIR**	96/497	1.22 (0.69,2.16)	110/754	0.85 (0.57,1.25)
	**ELIH**	89/482	1.03 (0.75,1.41)	108/746	1.02 (0.71,1.46)
	**ELIR**	89/482	1.49 (0.97,2.28)	108/746	0.73 (0.46,1.16)
**Prostate Cancer-Specific Mortality**					
**Dietary indices (per SD increase)[a]**					
**Healthy deitary indices**	**aMED**	47/497	1.64 (0.83,3.24)	50/754	0.71 (0.42,1.20)
	**AHEI-2010**	47/497	1.79 (0.85,3.76)	50/754	0.71 (0.40,1.24)
	**HEI-2015**	47/497	2.50 (1.05,5.96)	50/754	0.76 (0.46,1.26)
	**DASH**	47/497	3.28 (1.35,7.96)	50/754	0.66 (0.38,1.14)
	**PDI**	47/497	1.13 (0.66,1.95)	50/754	0.67 (0.38,1.19)
	**hPDI**	47/497	1.83 (0.95,3.51)	50/754	0.64 (0.37,1.11)
**Adverse dietary indices**	**uPDI**	47/497	0.46 (0.22,0.97)	50/754	1.28 (0.77,2.14)
	**E-DII**	47/497	0.55 (0.27,1.12)	50/754	1.82 (1.04,3.17)
	**EDIP**	47/497	0.77 (0.34,1.74)	50/754	2.97 (1.12,7.87)
	**EDIH**	47/497	0.60 (0.28,1.27)	50/754	1.14 (0.64,2.03)
	**EDIR**	47/497	0.90 (0.41,2.00)	50/754	1.23 (0.61,2.47)
	**ELIH**	44/482	1.13 (0.73,1.76)	50/746	1.22 (0.76,1.95)
	**ELIR**	44/482	1.12 (0.62,2.02)	50/746	1.67 (0.81,3.46)

[a] Models were adjusted for age at diagnosis, education, family history of prostate cancer, total calories intake

[b] Results for Native Hawaiians were not shown due to limited sample size;

[c] The p-value for heterogeneity (p-het) was was estimated using the Restricted Maximum Likelihood (REML)

etastatic Prostate Cancer in the MEC by Race and Ethnicity (N = 2603)

Latino		White		Tot
Events/No. at risk	HR (95%CI)	Events/No. at risk	HR (95%CI)	Events/No. at risk
271/577	0.80 (0.64,1.01)	334/646	0.91 (0.77,1.09)	1346/2603
271/577	0.83 (0.68,1.01)	334/646	0.95 (0.81,1.12)	1346/2603
271/577	0.83 (0.68,1.02)	334/646	0.87 (0.74,1.02)	1346/2603
271/577	0.91 (0.74,1.11)	334/646	0.92 (0.78,1.09)	1346/2603
271/577	0.79 (0.65,0.96)	334/646	0.95 (0.80,1.11)	1346/2603
271/577	0.82 (0.67,1.01)	334/646	0.92 (0.77,1.08)	1346/2603
271/577	1.07 (0.88,1.30)	334/646	1.13 (0.96,1.33)	1346/2603
271/577	1.10 (0.90,1.35)	334/646	1.07 (0.90,1.27)	1346/2603
271/577	1.20 (0.93,1.54)	334/646	1.01 (0.82,1.25)	1346/2603
271/577	1.43 (1.13,1.82)	334/646	1.11 (0.92,1.34)	1346/2603
271/577	1.25 (0.96,1.63)	334/646	1.05 (0.87,1.27)	1346/2603
265/566	1.02 (0.85,1.23)	332/643	1.29 (1.11,1.50)	1317/2565
265/566	1.00 (0.81,1.24)	332/643	1.32 (1.07,1.63)	1317/2565
96/577	0.75 (0.51,1.10)	99/646	0.92 (0.65,1.31)	426/2603
96/577	0.69 (0.48,0.99)	99/646	1.07 (0.77,1.49)	426/2603
96/577	0.76 (0.53,1.09)	99/646	0.90 (0.66,1.24)	426/2603
96/577	0.78 (0.55,1.11)	99/646	0.94 (0.67,1.32)	426/2603
96/577	0.77 (0.56,1.04)	99/646	0.79 (0.56,1.13)	426/2603
96/577	0.83 (0.59,1.17)	99/646	0.90 (0.63,1.27)	426/2603
96/577	1.01 (0.72,1.40)	99/646	0.97 (0.70,1.36)	426/2603
96/577	1.21 (0.85,1.71)	99/646	1.22 (0.87,1.71)	426/2603
96/577	1.64 (0.98,2.73)	99/646	0.83 (0.51,1.35)	426/2603
96/577	2.28 (1.45,3.57)	99/646	1.21 (0.81,1.81)	426/2603
96/577	1.71 (1.03,2.86)	99/646	0.93 (0.59,1.47)	426/2603
95/566	1.30 (0.95,1.79)	98/643	1.50 (1.11,2.04)	414/2565
95/566	1.41 (1.00,1.98)	98/643	1.40 (0.90,2.18)	414/2565
39/577	0.97 (0.51,1.85)	48/646	0.80 (0.50,1.28)	197/2603
39/577	1.33 (0.76,2.34)	48/646	0.99 (0.63,1.56)	197/2603
39/577	0.97 (0.54,1.74)	48/646	0.67 (0.43,1.06)	197/2603
39/577	1.21 (0.69,2.12)	48/646	0.74 (0.45,1.21)	197/2603
39/577	0.96 (0.58,1.61)	48/646	1.09 (0.69,1.72)	197/2603
39/577	1.04 (0.61,1.77)	48/646	1.06 (0.68,1.65)	197/2603
39/577	0.97 (0.56,1.67)	48/646	1.02 (0.63,1.63)	197/2603
39/577	0.93 (0.52,1.66)	48/646	0.66 (0.38,1.13)	197/2603
39/577	2.19 (0.93,5.13)	48/646	1.18 (0.68,2.05)	197/2603
39/577	1.56 (0.77,3.17)	48/646	0.93 (0.55,1.55)	197/2603
39/577	2.35 (1.02,5.42)	48/646	1.15 (0.69,1.91)	197/2603
39/566	0.83 (0.49,1.41)	48/643	0.61 (0.34,1.10)	194/2565
39/566	0.96 (0.52,1.78)	48/643	0.86 (0.51,1.44)	194/2565

e at QX3, prostate cancer stage, grade, treatments, and other lifestyle factors not included in the scores;

method in a random-effects model to assess the variance between race and ethnicity groups.

© 2025 Wang A et al. *JAMA Network Open* .

al HR (95%CI)	p-het[c]
0.88 (0.80,0.96)	0.83
0.92 (0.85,1.00)	0.69
0.90 (0.83,0.97)	0.89
0.95 (0.88,1.03)	0.78
0.90 (0.83,0.97)	0.51
0.92 (0.85,1.00)	0.81
1.10 (1.02,1.18)	0.87
1.04 (0.96,1.13)	0.79
1.02 (0.92,1.12)	0.26
1.09 (1.00,1.20)	0.02
1.00 (0.91,1.09)	0.35
1.06 (0.98,1.15)	0.01
1.03 (0.94,1.13)	0.10
0.91 (0.77,1.06)	0.06
0.90 (0.78,1.05)	0.16
0.88 (0.76,1.01)	0.67
0.94 (0.81,1.08)	0.60
0.83 (0.72,0.96)	0.20
0.89 (0.77,1.02)	0.77
1.07 (0.93,1.22)	0.48
1.04 (0.89,1.21)	0.03
1.12 (0.92,1.36)	0.19
1.34 (1.13,1.58)	0.04
1.10 (0.92,1.33)	0.16
1.19 (1.04,1.37)	0.26
1.19 (1.00,1.42)	0.09
1.02 (0.80,1.29)	0.25
1.05 (0.85,1.30)	0.20
0.97 (0.78,1.20)	0.06
1.05 (0.84,1.31)	0.01
1.11 (0.89,1.37)	0.54
1.09 (0.88,1.35)	0.12
1.01 (0.82,1.24)	0.17
0.95 (0.76,1.19)	0.03
1.09 (0.83,1.43)	0.12
0.97 (0.75,1.24)	0.31
1.01 (0.79,1.30)	0.40
1.03 (0.84,1.26)	0.25
1.01 (0.79,1.29)	0.51

© 2025 Wang A et al. *JAMA Network Open* .

eTable 9. The Associations of Dietary Indices With Mortality Among Men With Nonmeta

		No. at risk	All-cause Mortality Events	Model 1 HR (95%CI)[a]	Model 2 HR (95%CI)[b]
**Lifestyle scores (per point increase)[a]**					
	**2015 PCa Behavior Score**	5520	3175	0.89 (0.84,0.95	0.91 (0.85,0.97
	**2021 PCa Behavior Score**	5372	3065	0.65 (0.59,0.72	0.66 (0.60,0.74
	**2021 PCa Behavior Score Including Diet**	5369	3027	0.67 (0.61,0.74	0.67 (0.61,0.74
**Dietary indices (per SD increase)[b]**					
**Healthy deitary indices**	**aMED**	5663	3384	0.85 (0.80,0.91	0.87 (0.80,0.95
	**AHEI-2010**	5663	3384	0.89 (0.83,0.94	0.92 (0.85,1.00
	**HEI-2015**	5663	3384	0.86 (0.81,0.91	0.89 (0.82,0.97
	**DASH**	5663	3384	0.89 (0.84,0.95	0.95 (0.88,1.03
	**PDI**	5663	3384	0.91 (0.85,0.96	0.90 (0.83,0.97
	**hPDI**	5663	3384	0.89 (0.84,0.95	0.92 (0.85,1.00
**Adverse dietary indices**	**uPDI**	5663	3384	1.12 (1.05,1.19	1.10 (1.02,1.19
	**EDIP**	5663	3384	1.04 (0.96,1.11	1.02 (0.93,1.12
	**EDIH**	5663	3384	1.10 (1.03,1.18	1.09 (1.00,1.20
	**EDIR**	5663	3384	1.01 (0.94,1.09	1.00 (0.91,1.09
	**ELIH**	5413	3063	1.08 (1.01,1.15	1.06 (0.97,1.15
	**ELIR**	5413	3063	1.05 (0.96,1.14	1.02 (0.92,1.13
	**E-DII**	5663	3384	1.09 (1.03,1.16	1.04 (0.96,1.13

[a] Models were adjusted for age at diagnosis, education, ethnicity, family history of prostate cancer, total calories

[b] Models were adjusted for age at diagnosis, education, ethnicity, family history of prostate cancer, total calories

© 2025 Wang A et al. *JAMA Network Open* .

astatic Prostate Cancer in the MEC, Accounting for Survival Bias (N = 5667)

CVD-related Mortality			Prostate cancer-specific mortality		
Events	Model 1 HR (95%CI)[a]	Model 2 HR (95%CI)[b]	Events	Model 1 HR (95%CI)[a]	Model 2 HR (95%CI)[b]
996	0.86 (0.77,0.9	0.89 (0.80,1.00)	476	0.94 (0.80,1.11	0.92 (0.78,1.09)
960	0.60 (0.50,0.7	0.62 (0.52,0.75)	462	0.92 (0.70,1.21	0.90 (0.68,1.19)
953	0.63 (0.54,0.7	0.64 (0.54,0.75)	455	0.91 (0.71,1.1	0.92 (0.71,1.18)
1067	0.88 (0.78,0.9	0.90 (0.77,1.06)	505	0.98 (0.82,1.1	1.01 (0.80,1.28)
1067	0.87 (0.78,0.9	0.90 (0.77,1.04)	505	1.03 (0.87,1.21	1.04 (0.84,1.29)
1067	0.85 (0.77,0.9	0.87 (0.75,1.01)	505	1.04 (0.89,1.21	0.96 (0.77,1.19)
1067	0.89 (0.80,0.9	0.93 (0.80,1.07)	505	1.08 (0.92,1.2	1.04 (0.83,1.30)
1067	0.88 (0.80,0.9	0.84 (0.73,0.97)	505	1.07 (0.92,1.2	1.11 (0.90,1.38)
1067	0.85 (0.77,0.9	0.88 (0.77,1.02)	505	1.03 (0.88,1.2	1.09 (0.88,1.35)
1067	1.12 (1.01,1.2	1.07 (0.93,1.23)	505	1.04 (0.89,1.2	1.02 (0.84,1.26)
1067	1.19 (1.04,1.3	1.12 (0.93,1.36)	505	0.85 (0.72,1.01	1.09 (0.84,1.42)
1067	1.28 (1.14,1.4	1.33 (1.13,1.57)	505	0.85 (0.72,1.0	0.97 (0.76,1.24)
1067	1.18 (1.03,1.3	1.11 (0.92,1.33)	505	0.83 (0.70,0.9	1.02 (0.79,1.31)
968	1.24 (1.11,1.3	1.18 (1.02,1.37)	459	1.02 (0.86,1.2	1.00 (0.81,1.25)
968	1.26 (1.09,1.4	1.18 (0.97,1.42)	459	0.96 (0.79,1.1	1.03 (0.79,1.33)
1067	1.09 (0.98,1.2	1.04 (0.90,1.21)	505	0.93 (0.80,1.0	0.96 (0.77,1.19)

intake at QX3, prostate cancer stage, grade, and treatments;

intake at QX3, prostate cancer stage, grade, treatments, and other lifestyle factors not included in the

© 2025 Wang A et al. *JAMA Network Open* .

scores.

© 2025 Wang A et al. *JAMA Network Open* .
