# Supplementary material for: Race and Ethnicity, Lifestyle, Diet, and Survival in Patients With Prostate Cancer
Source: JAMA Netw Open. 2025 Feb 26;8(2):e2460785. doi: 10.1001/jamanetworkopen.2024.60785 (PMC11866029; doi:10.1001/jamanetworkopen.2024.60785)
Supplement: Supplement 3. — Data Sharing Statement [file jamanetwopen-e2460785-s003.pdf]

Data Sharing Statement

Wang. Race and Ethnicity, Lifestyle, Diet, and Survival in Patients With Prostate Cancer. *JAMA Netw Open*. Published February 20, 2025. doi:10.1001/jamanetworkopen.2024.60785

Data
Data available: Yes
Data types: Deidentified participant data
How to access data: The data underlying this analysis were provided by the Multiethnic Cohort Study (MEC) under data use agreement. The data used in this study are available in dbGaP (phs002183.v1.p1).
When available: With publication

Supporting Documents
Document types: None

Additional Information
Who can access the data: Investigators must be permanent employees of their institution at a level equivalent to a tenure-track professor or senior scientist with responsibilities that most likely include laboratory administration and oversight. Researchers whose proposed use of the data has been approved.
Types of analyses: for research purpose
Mechanisms of data availability: After approval of a proposal and with a signed data access agreement
Any additional restrictions: Compliance with dbGaP dataset access requirements.
